# Increasing the source/sink ratio in *Vitis vinifera *(cv Sangiovese) induces extensive transcriptome reprogramming and modifies berry ripening

**DOI:** 10.1186/1471-2164-12-631

**Published:** 2011-12-23

**Authors:** Chiara Pastore, Sara Zenoni, Giovanni Battista Tornielli, Gianluca Allegro, Silvia Dal Santo, Gabriele Valentini, Cesare Intrieri, Mario Pezzotti, Ilaria Filippetti

**Affiliations:** 1 Department of Fruit Tree and Woody Plant Science, University of Bologna, Viale Fanin, 46, 40126, Bologna, Italy; 2 Department of Biotechnology, University of Verona, Strada Le Grazie 15, 37134, Verona, Italy

## Abstract

**Background:**

Cluster thinning is an agronomic practice in which a proportion of berry clusters are removed from the vine to increase the source/sink ratio and improve the quality of the remaining berries. Until now no transcriptomic data have been reported describing the mechanisms that underlie the agronomic and biochemical effects of thinning.

**Results:**

We profiled the transcriptome of *Vitis vinifera *cv. Sangiovese berries before and after thinning at veraison using a genome-wide microarray representing all grapevine genes listed in the latest V1 gene prediction. Thinning increased the source/sink ratio from 0.6 to 1.2 m^2 ^leaf area per kg of berries and boosted the sugar and anthocyanin content at harvest. Extensive transcriptome remodeling was observed in thinned vines 2 weeks after thinning and at ripening. This included the enhanced modulation of genes that are normally regulated during berry development and the induction of a large set of genes that are not usually expressed.

**Conclusion:**

Cluster thinning has a profound effect on several important cellular processes and metabolic pathways including carbohydrate metabolism and the synthesis and transport of secondary products. The integrated agronomic, biochemical and transcriptomic data revealed that the positive impact of cluster thinning on final berry composition reflects a much more complex outcome than simply enhancing the normal ripening process.

## Background

Many agronomic practices are employed to maximize grape berry quality in the highly competitive wine industry, including the control of bud load during winter pruning and cluster thinning during berry development. Cluster thinning acts directly to increase the source/sink balance of grapevine plants and the technique is used to prevent overcropping in varieties characterized by excessive bud fertility or in areas where reduced yield is a prerequisite for high-quality wine production. Under such conditions, cluster thinning is performed to obtain a leaf area/yield ratio of 0.8-1.2 m^2^/kg. Below this threshold value several authors [1,2] reported a positive correlation between berry juice soluble sugars and leaf area/crop weight ratio. Similar results have been achieved following cluster thinning in different varieties and sites [3-7]. Thinning also increases the anthocyanin content of berries, which is an important quality determinant of red wines [8-10]. The anthocyanin composition is also affected, e.g. cluster thinning induced the accumulation of 3',4'-substituted anthocyanins in Sangiovese and Nebbiolo varieties [3,5].

Although it is well known that many different factors influence flavonoid and anthocyanin biosynthesis [11], sugar is likely to play a prominent role because of the concomitant accumulation of soluble solids in the berry flesh and anthocyanins in the skin of red grape varieties. This relationship was first proposed by Pirie and Mullins [12], who suggested that the sugar content of red berry flesh could regulate anthocyanin production, and this was supported by in vitro experiments showing an increase in phenylalanine ammonia-lyase (*PAL*) activity [13]. Increased anthocyanin accumulation after treatment with sucrose and other sugars has been already demonstrated in grapevine and in a variety of other plant species [14-17]. Several sugars have been shown to induce genes encoding enzymes in the anthocyanin biosynthesis pathway, such as chalcone synthase (*CHS*), dihydroflavonol reductase (*DFR*), leucoanthocyanidin dioxygenase (*LDOX*) [18,19] and flavanone 3-hydroxylase (*F3H*) [20]. Sucrose boxes have been identified in the promoters of some of these genes [18,19].

Little is currently known about the regulation of gene expression when the source/sink ratio is deliberately altered in the field. We report the first transcriptomic analysis (integrated with agronomic and biochemical data) aiming to determine the mechanisms that control Sangiovese berry composition by comparing gene expression profiles of thinned and control vines. Berry transcriptional profiles were analyzed during ripening using the most comprehensive grapevine microarray available to date, representing 29,549 genes from the most recent 12X grapevine V1 gene prediction http://srs.ebi.ac.uk/. We observed substantial transcriptomic remodeling in berries from thinned vines which became evident by 2 weeks post-treatment and persisted until ripening, with particular impact on genes involved in carbohydrate metabolism, flavonoid biosynthesis and transport. The results from these studies provide insight into the molecular basis of berry ripening induced by vineyard management techniques.

## Results

### The effect of cluster thinning on yield and berry ripening

Cluster thinning (CT) was carried out to remove approximately 50% of the bunches on each vine, leaving approximately eight clusters per vine in comparison with 16 on control (C) plants, thus reducing the yield by ~54% (Table 1). The average bunch and berry weight remained the same in CT and C plants (Table 1).

**Table 1:** Influence of cluster thinning (CT) on yield component and berry composition at harvest.

	Yield/ vine (kg)	Cluster/ vine (n)	Cluster weight (g)	Berry weight (g)	Leaf area/ vine (m^2^)	Leaf area/ yield (m^2^/kg)	°Brix	TA (g/L)	pH
C	6.3 a	16 a	386	2.37	3.84	0.6 b	20.8 b	7.6 a	3.4 b

CT	2.9 b	8 b	353	2.24	3.43	1.2 a	22.7 a	6.8 b	3.5 a

Significance^zy^	*	*	ns	ns	ns	*	*	*	*

^z ^Means separated within columns by the Student-Newman-Keuls test (n = 12 except for Brix, TA and pH were n = 3).

^y ^*, significant at p ≤ 0.05; ns, not significant.

**Figure 1:**
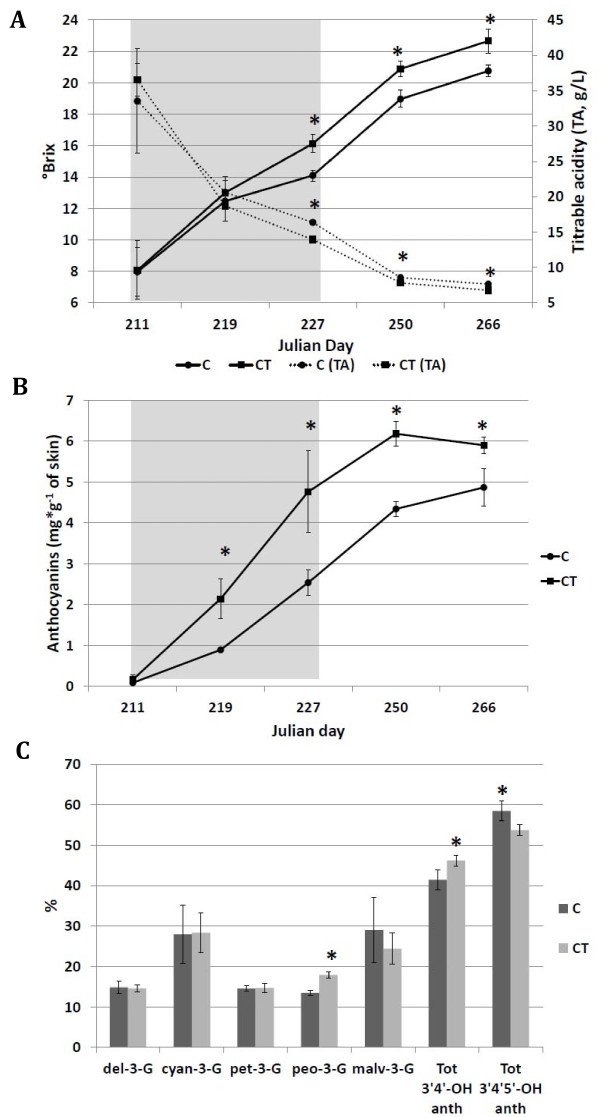
**Agronomic effects of cluster thinning**. (A) Accumulation of soluble sugars (°Brix) and titratable acidity (TA) in berries from C and CT vines (n = 3). (B) Anthocyanin concentration during ripening and (C) anthocyanin composition at harvest in berry skins from C and CT vines (n = 3). Bars represent ± SE. Asterisks indicate significant differences between the treatments at the same date using the Student-Newman-Keuls test (*P < 0.05). Gray background represents the veraison phase.

The leaf area per vine was similar in CT and C plants at harvest, indicating that cluster thinning increased the leaf area/yield ratio from 0.6 m^2^/kg in C plants to 1.2 m^2^/kg in CT plants (Table 1). CT berries also accumulated more total soluble solids than C berries from full veraison (8 d after cluster thinning) until harvest, and were less acidic and had higher °Brix values than controls at harvest (Figure 1A and Table 1).

The anthocyanin content of berry skin was analyzed by HPLC over the same period, showing that anthocyanins accumulated more rapidly in CT berries compared to controls (Figure 1B), and the difference in anthocyanin content between the two samples was already significantly higher by the second sampling date (JD 219) corresponding to full veraison, and gradually declined but was still evident at harvest (Figure 1B). HPLC analysis also revealed that the increase in total anthocyanins was not evenly distributed among the five main glucosylated species that characterize the Sangiovese cultivar (Figure 1C). We observed a significant increase in the levels of 3'4'-OH anthocyanins associated to cluster thinning, which modified the anthocyanin profile of CT berries, particularly increasing levels of the glucosylated form of peonidin compared to C berries.

### Transcriptional modulation induced by cluster thinning

To investigate the molecular changes that take place in response to cluster thinning, we carried out a comparative microarray analysis of CT and C berries at time points JD 211, 227 and 266, corresponding to the beginning of veraison (BV), the end of veraison (EV) and harvest (H).

**Figure 2:**
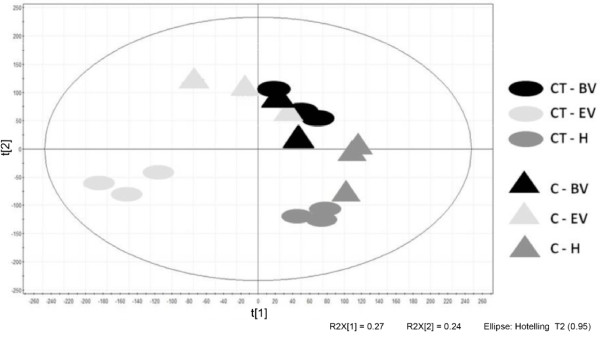
**Principal component analysis (PCA) shows that the most severe changes are at the end of veraison**. Biological replicates relative to time point beginning of veraison (BV), end of veraison (EV) and harvest (H) are represented as circles for CT and as triangles for C.

Principal Component Analysis (PCA) of the global transcriptomic data revealed enough uniformity among the three biological replicates to defined associations between treatments (Figure 2). The two principal components, explaining about the 50% of the overall variance, allowed us to clearly separate C and CT at the EV stage, whereas the separation was less clear-cut at the BV and H stages suggesting that the main transcriptomic changes induced by cluster thinning occurred at the EV stage.

**Figure 3:**
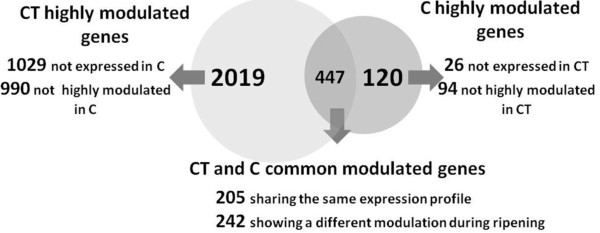
**Differentially expressed genes during ripening in CT and C berries**. Transcripts are divided into three different gene datasets according to their high modulation in CT, in C or in both. The number of CT highly modulated genes either not expressed in control berries or expressed but not highly modulated is shown. Similarly the number of C highly modulated genes either not highly expressed in CT berries or expressed but not highly modulated is shown. The number of common modulated transcripts with equal or different modulation is specified.

To identify the gene expression profiles with the greatest contribution to the differences between the C and CT transcriptomes, a multiclass comparison analysis was carried out using Significance Analysis of Microarray (SAM) with a false discovery rate (FDR) of 2% (TMev 4.3). We identified 1626 genes modulated during C berry development and 6033 modulated during CT berry development, with a fold change ≥ 2 in at least one comparison (Additional File 1). To evaluate the principal modifications triggered by cluster thinning, we focused on genes with a fold change ≥ 5, narrowing the analysis to 567 genes modulated during C berry development and 2466 genes modulated during CT berry development. A comparison of these datasets indicated three different sets of modulated transcripts. The first grouped 447 genes modulated in both treatments, the second grouped 2019 genes modulated only in CT berries and the third grouped 120 genes modulated only in C berries (Figure 3). For convenience, genes that showed less than a five-fold change in expression were described as 'not highly modulated'.

Clustering analysis using Pearson's correlation distance divided the common, CT highly modulated and C highly modulated transcripts (Tables 2, 3 and 4) into eight groups representing the minimum number of profiles required to describe the three sampling time points. Clusters 1-4 represent genes that are downregulated during at least one analyzed time point compared to the BV stage, whereas clusters 5-8 represent genes that are upregulated during at least one analyzed time point compared to the BV stage.

**Table 2:** Cluster distribution of genes highly modulated both in C and CT berries.

Cluster number	Expression profile	Common modulated genes	
		
		C	CT
**1**	****	5	8

**2**	****	80	179

**3**	****	216	90

**4**	****	14	40

**5**	****	1	2

**6**	****	40	44

**7**	****	78	70

**8**	****	13	14

Analysis was performed separately for C and CT berries at the beginning of veraison (BV), end of veraison (EV) and harvest (H). The expression profiles of the 447 common modulated genes during berry ripening were clustered in eight groups obtained by the k-means method using Pearson's correlation distance. The representative profile and the number of genes for each treatment in every cluster are indicated.

**Table 3:** Distribution of 192 annotated transcripts (common to C and CT berries but differentially modulated during ripening) into eight clusters according to the gene expression trends.

		CLUSTER 1	CLUSTER 2	CLUSTER 3	CLUSTER 4	CLUSTER 5	CLUSTER 6	CLUSTER 7	CLUSTER 8
		
									
**Carbohydrate metabolic process**	**C**	0	0	3	0	0	2	0	0
	
	**CT**	0	3	0	0	0	0	2	0

**Cell wall organization or biogenesis**	**C**	0	4	6	0	0	1	0	0
	
	**CT**	0	6	3	1	0	0	1	0

**Cellular amino acid and derivative metabolic process**	**C**	0	2	5	0	0	1	1	0
	
	**CT**	1	5	1	1	0	0	0	1

**Cellular homeostasis**	**C**	0	2	3	0	0	1	0	0
	
	**CT**	0	3	0	2	0	0	1	0

**Cellular process**	**C**	1	2	6	0	0	2	2	1
	
	**CT**	0	6	1	2	0	2	2	1

**Developmental process**	**C**	0	1	4	1	0	3	0	1
	
	**CT**	0	5	0	1	0	1	2	1

**Generation of precursor metabolites and energy**	**C**	0	0	27	1	0	0	0	0
	
	**CT**	1	25	0	2	0	0	0	0

**Hormone metabolic process**	**C**	0	1	4	0	0	0	0	0
	
	**CT**	0	4	1	0	0	0	0	0

**Lipid metabolic process**	**C**	0	2	4	0	0	1	0	1
	
	**CT**	0	2	1	3	0	1	1	0

**Nitrogen compound metabolic process**	**C**	0	0	1	0	0	0	0	0
	
	**CT**	0	1	0	0	0	0	0	0

**Nucleic acid metabolic process**	**C**	0	0	2	0	0	0	0	0
	
	**CT**	0	2	0	0	0	0	0	0

**Protein metabolic process**	**C**	0	0	0	0	0	0	0	0
	
	**CT**	0	0	0	0	0	0	0	0

**Response to hormone stimulus**	**C**	0	0	9	0	0	0	1	0
	
	**CT**	0	7	0	2	0	1	0	0

**Response to stress**	**C**	0	1	7	0	0	2	4	2
	
	**CT**	1	7	1	0	0	2	2	3

**Secondary metabolic process**	**C**	0	3	3	2	0	0	10	0
	
	**CT**	0	4	2	2	0	9	0	1

**Signal transduction**	**C**	0	2	9	0	0	2	0	0
	
	**CT**	0	8	1	2	0	0	2	0

**Transcription**	**C**	0	1	11	0	0	1	2	0
	
	**CT**	0	9	0	3	0	2	1	0

**Transport**	**C**	0	3	8	3	0	3	3	1
	
	**CT**	0	11	0	3	0	3	3	1

**TOTAL DIFFERENTIAL MODULATED GENES per CLUSTER**	**C**	**1**	**24**	**112**	**7**	**0**	**19**	**23**	**6**
	
	**CT**	**3**	**108**	**11**	**24**	**0**	**21**	**17**	**8**

In each cluster, transcripts are divided according to their functional categories.

**Table 4:** Cluster distribution of highly modulated genes in CT and C berries.

			**CT**			**C**	
Cluster number	Expression profile	Total genes	Genes not expressed in C	Genes not highly modulated in C	Total genes	Genes not expressed in CT	Genes not highly modulated in CT
**1**	****	404	23	381	3	0	3
**2**	****	251	30	221	21	5	16
**3**	****	58	4	54	16	1	15
**4**	****	172	25	147	1	0	1
**5**	****	357	303	54	5	2	3
**6**	****	196	164	32	38	7	31
**7**	****	89	54	35	34	10	24
**8**	****	492	426	66	2	1	1

Expression profiles of the 2019 CT and 120 C highly modulated genes during berry ripening were clustered in eight groups obtained by the k-means method using Pearson's correlation distance. The representative profile and the number of genes in each cluster are indicated. For each cluster, the CT highly modulated genes that are expressed without high modulation or not expressed at all in control berries are indicated, and similarly the C highly modulated genes that are expressed without high modulation in CT berries or not expressed at all are indicated.

**Figure 4:**
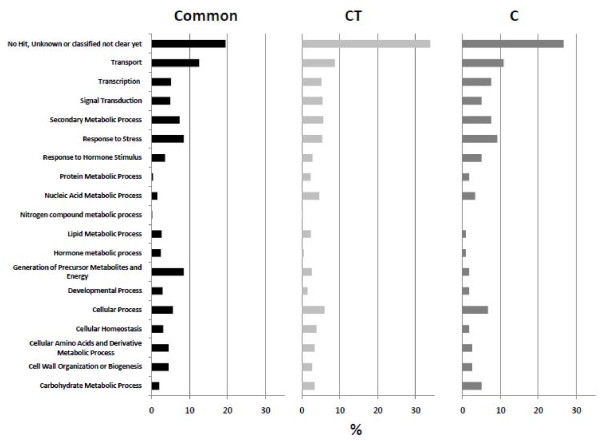
**Distribution of common, CT and C highly modulated transcripts into 18 GO functional categories**. Transport: GO:0006810; Transcription: GO:0006350; Signal Transduction: GO:0007165; Secondary Metabolic Process: GO:0019748; Response to Stress: GO:0006950; Response to Hormone Stimulus: GO:0009725; Protein Metabolic Process: GO:0019538; Nucleic Acid Metabolic Process: GO:0090304; Nitrogen Compound Metabolic Process: GO:0006807; Lipid Metabolic Process: GO:0006629; Hormone Metabolic Process: GO:0042445; Generation of precursor metabolites and energy: GO:0006091; Developmental Process: GO:0032502; Cellular Process: GO:0009987; Cellular Homeostasis: GO:0019725; Cellular amino acid and derivative metabolic process: GO:0006519; Cell wall organization or biogenesis: GO:0071554; Carbohydrate metabolic process: GO: 0005975. Percentages were calculated on the total number of modulated genes for each dataset.

All the transcripts were annotated against the V1 version of the 12X draft annotation of the grapevine genome http://genomes.cribi.unipd.it/DATA/ allowing 70% of the modulated genes to be identified (Additional File 2). To investigate the functional distribution of commonly and specifically modulated transcripts, we distributed them into 18 Gene Ontology (GO) functional categories and determined the percentage of genes in each category for each of the three data sets (Figure 4). The most represented functional categories, shared among the three datasets, were "Transport", "Transcription", "Secondary Metabolic Process", "Response to Stress" and "Cellular Process", which included the main genes involved in the physiology of berry ripening. The functional distribution of modulated genes was similar in the CT and C specific gene sets, suggesting that the CT treatment has a widespread effect on transcription rather than impacting on a specific functional category. The results for the common, CT highly modulated and C highly modulated genes are discussed in more detail below.

### Common modulated genes

The first gene set contains 447 genes that are modulated in both C and CT berries, representing 79% of the genes modulated in C berries and 22% of those modulated in CT berries. Approximately equal numbers of genes for each treatment were upregulated in comparison to the BV stage and were therefore distributed into clusters 5-8 (132 for C berries and 130 for CT berries, Table 2). Similarly, there were approximately equal numbers of downregulated genes distributed into clusters 1-4 (315 for C berries and 317 for CT berries) although the nature of the distribution was distinct (Table 2). In particular, there were more genes downregulated throughout ripening (cluster 2) and specifically between BV to EV (cluster 4) in CT berries, whereas there were more genes downregulated specifically between EV and H (cluster 3) in C berries. Interestingly, very few common genes were detected in clusters 1 and 5, which represent EV-specific downregulation (1) and upregulation (5) in both treatments.

We identified 205 genes with similar expression profiles in both treatments and 242 with expression profiles that differed between C and CT berries during ripening (Figure 3, Additional File 2). The cluster distribution for the 192 genes with successful functional annotations belonging to the latter group is shown in Table 3.

Many genes belonging to "Generation of Precursor Metabolites and Energy", the most representative functional category, and to several others such as "Carbohydrate Metabolic Process", "Cell Wall Organization or Biogenesis", "Hormone Metabolic Process", "Response to Hormone Stimulus", "Response to Stress" and "Transport", were allocated to cluster 3 in C berries (downregulated specifically between EV and H) but shifted to cluster 2 in CT berries (downregulated throughout ripening). This pattern was particularly evident among the 24 genes with a role in photosynthesis, the six involved in the cell wall changes associated with berry softening (e.g. pectate lyase, pectinesterase and xyloglucan endotransglucosylase), the three involved in sugar metabolism (fructose-bisphosphate aldolase, the vacuolar invertase *GIN1 *and galactinol synthase) and the two involved in ethylene biosynthesis, i.e. 1-aminocyclopropane-1-carboxylic acid synthase (*ACS*) and 1-aminocyclopropane-1-carboxylic acid oxidase (*ACO*). These data are presented in Additional File 2. For the vacuolar invertase *GIN1 *(VIT_160022g00670), the expression profiles in C and CT berries were confirmed by real-time RT-PCR (Additional File 3).

We also noted several genes that were allocated to cluster 7 in C berries (upregulated specifically between EV to H) but to cluster 6 in CT berries (upregulated throughout ripening) (Table 3). These genes were predominantly involved in secondary metabolic processes, e.g. five *PAL *isogenes (VIT_00s2508g00010, VIT_16s0039g01120, VIT_16s0039g01320, VIT_16s0039g01300, VIT_00s2849g00010) and two stilbene synthases (*STSs*, VIT_16s0100g01070, VIT_16s0100g00850). We confirmed the expression profile of one *PAL *(VIT_16s0039g01120) in C and CT berries by real-time RT-PCR (Additional File 3).

In addition, several of the 205 genes whose expression profile did not change qualitatively after cluster thinning showed marked quantitative differences in expression (Additional File 4). In particular, we observed stronger downregulation of several genes involved in photosynthesis, cell wall metabolism, stress responses and hormone metabolism in CT berries. Notable examples included genes involved in the dynamic remodeling of cell wall polysaccharides, such as a xyloglucan endotransglucosylase/hydrolase (VIT_00s0386g00050), which was downregulated 10-fold more strongly in CT berries compared to controls, and genes related to hormone metabolism, such as a cytokinin dehydrogenase (VIT_18s0001g13200).

Whereas many common genes were downregulated more strongly in CT berries compared to controls, very few were induced more strongly after cluster thinning. However, examples included galactinol synthase (VIT_07s0005g0970), which is an important regulator of carbon partitioning, as well as a *STS *(VIT_09s0018g01490) and a *PAL *involved in the phenylpropanoid/flavonoid pathway (VIT_16s0039g01170). Remarkably, the glutathione-S-transferase *VvGST4 *(VIT_04s0079g00690), whose involvement in berry ripening has been reported previously [21], was upregulated throughout ripening in CT berries compared to controls. We validated the expression profile of one gene in this group by real-time RT-PCR, the flavonol synthase VIT_18s0001g03430 (Additional File 3).

### Genes highly modulated in response to cluster thinning

The second group of modulated genes comprised 2019 CT highly modulated transcripts that could be divided into eight clusters according to their expression profiles (Table 4). Approximately equal numbers of genes were downregulated (885, clusters 1-4) and upregulated (1134, clusters 5-8). Unlike the cluster distribution of the common genes discussed above, many CT highly modulated genes were assigned to clusters 1 and 5, and most were assigned to clusters 1, 2, 4, 5, 6 and 8, characterized by prompt modulation in response to cluster thinning (i.e. changes already visible at EV). This suggests that physiological and biochemical changes in the berry almost occur immediately after the treatment. We investigated the expression of the CT highly modulated genes in C berries (Table 4), which allowed us to distinguish between those not expressed in C berries and those expressed but not highly modulated in C berries. Among the 885 genes grouped in clusters 1-4, 82 were not expressed in the controls and 803 were expressed but not highly modulated during ripening. The expression of 82 genes in CT berries but not in C berries at the earliest sampling stage is unlikely to reflect a response to treatment and is probably the consequence of inter-vine variability at the BV stage. Certain agronomic parameters such as titratable acidity and soluble sugar content showed higher standard deviations at BV than the subsequent stages (Figure 1A), confirming that samples collected at BV could be more heterogeneous than those collected later. This could likewise explain the presence of C highly downregulated transcripts that are not expressed in CT berries (Table 4). Surprisingly, a significantly majority of the 1134 genes in clusters 5-8 were not expressed in C berries, while only a minority was expressed but not highly modulated in C berries.

The 2019 CT highly modulated genes were assigned to 18 functional categories as shown in Figure 4. Given the major influence of carbohydrate metabolism, secondary metabolism and the transport of carbohydrate and secondary metabolites on berry quality traits, we will focus on genes in the categories "Carbohydrate Metabolic Process", "Secondary Metabolism" and "Transport", which represent 18% of all the genes modulated in CT berries following the treatment.

#### Genes belonging to the "Carbohydrate Metabolic Process" category

We identified 68 CT highly modulated genes involved in carbohydrate metabolism. These are listed in Table 5, which provides the Gene_ID, annotation, metabolic process, cluster assignment, expression in C berries, and the EV/BV and H/BV fold change. We found that 28 of these genes grouped in clusters 1-4 and 40 (including 33 not expressed in control berries) grouped in clusters 5-8. The modulated genes are involved in various primary metabolic pathways, including sucrose and starch metabolism, glycolysis, the pentose phosphate pathway and the Krebs cycle, indicating a large-scale reprograming of carbohydrate metabolism in response to cluster thinning. This includes the downregulation of two invertases (VIT_18s0072g01040 and VIT_00s2527g00010) and one sucrose-phosphate synthase (VIT_05s0029g01140) probably representing the decline in photosynthesis during berry ripening. The downregulation of VIT_00s2527g00010 was confirmed by real time RT-PCR (Additional File 3).

**Table 5:** CT highly modulated genes in the "Carbohydrate metabolic process" functional category.

Gene_ID	Description	Metabolic Process	Profile	Expression in C	FC EV/BV	FC H/BV
VIT_18s0072g00770	fructose-1,6-bisphosphatase, cytosolic	Gluconeogenesis	1	**E**	-12.1	-4.3
VIT_07s0205g00090	glycogen synthase 2	Glycogenesis	1	**E**	-6.6	-1.2
VIT_18s0001g15580	glycogenin glucosyltransferase (glycogenin)	Glycogenesis	1	**E**	-6.5	-2.1
VIT_06s0004g06920	fructose-6-phosphate-2-kinase	Glycolysis/Gluconeogenesis	1	**E**	-6.6	-1.6
VIT_06s0004g05900	Phosphopyruvate hydratase.	Glycolysis/Gluconeogenesis	1	**E**	-5.6	-1.5
VIT_01s0011g00250	6-phosphogluconolactonase	Pentose phosphate pathway	1	**E**	-5.9	-1.5
VIT_14s0030g01900	ribose-5-phosphate isomerase	Pentose phosphate pathway/Calvin cycle	1	**E**	-7.8	-2.1
VIT_02s0087g00440	Beta-amylase 8	Starch metabolism	1	**E**	-5.4	-1.7
VIT_17s0000g07680	alpha-N-aEtylglucosaminidase	Sugar metabolism	1	**E**	-5.09	-1.37
VIT_18s0072g01040	Invertase, neutral/alkaline	Sugar metabolism	1	**E**	-5.3	-1.9
VIT_00s1530g00010	stachyose synthase precursor	Sugar metabolism	1	**E**	-5.7	-3.7
VIT_05s0029g01140	sucrose-phosphate synthase	Sugar metabolism	1	**E**	-6.5	-0.5
VIT_11s0065g00150	glycogen synthase	Glycogenesis	1	**NE**	-5.1	-1.1
VIT_03s0038g04570	ADP-glucose pyrophosphorylase large subunit 1	Starch metabolism	1	**NE**	-8.3	-2.2

VIT_04s0023g03010	fructose-bisphosphate aldolase, chloroplast precursor	Glycolysis	2	**E**	-4.0	-5.6
VIT_14s0068g00680	glyEraldehyde-3-phosphate dehydrogenase A, chloroplast precursor	Glycolysis	2	**E**	-6.7	-9.8
VIT_14s0108g00270	aldose 1-epimerase	Glycolysis/Gluconeogenesis	2	**E**	-1.9	-5.2
VIT_03s0088g01190	malate dehydrogenase, glyoxysomal precursor	Malic acid metabolism	2	**E**	-1.6	-5.5
VIT_08s0007g01570	fructose 1,6-bisphosphatase	Pentose phosphate pathway	2	**E**	-6.9	-13.1
VIT_11s0078g00310	isoamylase-type starch-debranching enzyme 1	Starch metabolism	2	**E**	-3.3	-7.6
VIT_05s0049g01130	aldo/keto reductase	Sugar metabolism	2	**E**	-6.2	-7.1
VIT_00s2527g00010	beta-fructosidase/invertase	Sugar metabolism	2	**E**	-3.7	-5.2
VIT_14s0060g00740	galactinol synthase [Vitis riparia]	Sugar metabolism	2	**NE**	-5.3	-8.1

VIT_05s0094g00930	Phosphoglucomutase/phosphomannomutase C terminal	Carbohydrate metabolic proEss	4	**E**	-10.6	-10.3
VIT_08s0007g07600	pyruvate kinase, cytosolic isozyme	Glycolysis	4	**E**	-6.9	-5.5
VIT_15s0048g00370	transketolase, chloroplast precursor	Glycolysis	4	**E**	-12.5	-9.9
VIT_05s0020g02310	pyruvate, orthophosphate dikinase	Pyruvate metabolism	4	**E**	-15.0	-9.7
VIT_13s0019g02330	GDP-mannose pyrophosphorylase (GMP1)	Sugar metabolism	4	**E**	-4.7	-5.2

VIT_18s0089g00590	GlyEraldehyde-3-phosphate dehydrogenase, cytosolic	Glycolysis	5	**NE**	5.1	0.6
VIT_10s0003g05550	carbohydrate oxidase	Pentose phosphate pathway	5	**NE**	16.3	0.8
VIT_14s0060g00730	galactinol synthase	Sugar metabolism	5	**NE**	10.2	1.2

VIT_05s0062g00990	aldo/keto reductase AKR	Aldehyde detoxification pathways (oxidative stress)	6	**E**	4.3	6.0
VIT_02s0025g01560	UDP-glucose 4-epimerase GEPI48	Sugar metabolism	6	**E**	6.2	7.6
VIT_06s0004g02060	aldehyde dehydrogenase 3B1	Aldehyde detoxification pathways (oxidative stress)	6	**NE**	2.3	7.2
VIT_08s0007g01010	aldo/keto reductase	Aldehyde detoxification pathways (oxidative stress)	6	**NE**	10.6	16.8
VIT_13s0064g01420	succinate dehydrogenase [ubiquinone] flavoprotein subunit	Citric acid cycle	6	**NE**	4.5	7.0
VIT_07s0005g00440	pyruvate kinase	Glycolysis	6	**NE**	5.7	15.3
VIT_00s0233g00030	trehalose-6-phosphate phosphatase	Stress toleranE	6	**NE**	2.2	7.9

VIT_02s0241g00180	UDP-D-GLUCURONATE 4-EPIMERASE 5 GAE5	Carbohydrate metabolic proEss	7	**E**	1.4	13.8
VIT_14s0036g01210	trehalose 6-phosphate synthase	Stress toleranE	7	**E**	0.8	5.9
VIT_13s0019g04370	phosphoglucomutase/phosphomannomutase	Carbohydrate metabolic proEss	7	**NE**	1.6	9.6
VIT_00s0173g00110	Trehalose-phosphatase	Starch and sucrose metabolism	7	**NE**	1.4	8.2
VIT_11s0037g00710	trehalose-phosphate phosphatase	Starch and sucrose metabolism	7	**NE**	1.4	13.9
VIT_03s0063g00410	Alpha-amylase	Starch metabolism	7	**NE**	1.2	7.5

VIT_04s0044g01130	alcohol dehydrogenase [Vitis vinifera]	Fermentative metabolism	8	**E**	7.1	2.4
VIT_04s0044g01120	alcohol dehydrogenase [Vitis vinifera]	Fermentative metabolism	8	**E**	12.9	5.1
VIT_07s0005g03360	malate dehydrogenase, cytosolic	Malic acid metabolism	8	**E**	5.8	4.3
VIT_19s0085g01240	gamma hydroxybutyrate dehydrogenase-like protein	Butanoate metabolism	8	**NE**	7.9	5.6
VIT_15s0046g00910	serine/threonine protein phosphatase 1; PP1	Carbohydrate metabolic proEss	8	**NE**	7.6	3.0
VIT_04s0008g02300	pyruvate dehydrogenase E1 beta subunit	Fermentative metabolism	8	**NE**	11.3	11.2
VIT_07s0205g00070	phosphoenolpyruvate carboxykinase	Gluconeogenesis	8	**NE**	41.7	17.4
VIT_14s0171g00440	GlyEraldehyde-3-phosphate dehydrogenase GAPC3, cytosolic	Glycolysis	8	**NE**	27.4	3.8
VIT_01s0137g00090	aldehyde dehydrogenase (NAD+)	Glycolysis/Gluconeogenesis	8	**NE**	5.9	7.2
VIT_01s0137g00080	aldehyde dehydrogenase (NAD+)	Glycolysis/Gluconeogenesis	8	**NE**	14.0	12.7
VIT_07s0005g00430	pyruvate kinase	Glycolysis/Gluconeogenesis	8	**NE**	6.9	5.7
VIT_07s0005g03350	malate dehydrogenase, cytosolic	Malic acid metabolism	8	**NE**	7.7	2.7
VIT_03s0038g00040	NADP dependent malic enzyme	Malic acid metabolism	8	**NE**	21.5	3.2
VIT_16s0039g01050	NADP dependent malic enzyme	Malic acid metabolism	8	**NE**	34.2	9.6
VIT_16s0039g00580	NADP dependent malic enzyme	Malic acid metabolism	8	**NE**	8.1	6.2
VIT_15s0045g00190	NADP dependent malic enzyme	Malic acid metabolism	8	**NE**	40.8	6.5
VIT_04s0008g00180	NADP-dependent malic enzyme	Malic acid metabolism	8	**NE**	14.0	3.0
VIT_16s0013g01670	6-phosphogluconate dehydrogenase, cytosolic	Pentose phosphate pathway	8	**NE**	34.3	27.4
VIT_02s0012g03060	6-phosphogluconate dehydrogenase, decarboxylating	Pentose phosphate pathway	8	**NE**	13.2	12.8
VIT_05s0051g00010	beta-amylase 1	Starch metabolism	8	**NE**	8.1	5.4
VIT_16s0022g00740	granule-bound starch synthase Ib precursor	Starch metabolism	8	**NE**	34.1	36.4
VIT_00s0131g00420	Isoamylase isoform 3	Starch metabolism	8	**NE**	6.4	3.2
VIT_00s1562g00010	Sucrose synthase 2	Sugar metabolism	8	**NE**	19.9	3.2
VIT_18s0075g00330	sucrose-phosphate synthase	Sugar metabolism	8	**NE**	11.4	8.2

For each gene (Gene_ID) the annotation (Description), the function (Metabolic Process), the cluster number (Profile), the expression in C (E = expressed but not highly modulated; NE = not expressed) and the Fold Change (FC) referred to BV are reported.

We identified several genes involved in the synthesis and/or degradation of sugars and starch that were specifically induced by cluster thinning, including two genes involved in sucrose re-synthesis (the sucrose synthase VIT_00s1562g00010, and the sucrose-phosphate synthase VIT_18s0075g00330), four genes involved in glycolysis (the glyceraldehyde-3-phosphate dehydrogenases VIT_14s0171g00440 and VIT_18s0089g00590, and the pyruvate kinases VIT_07s0005g00440 and VIT_07s0005g00430), four involved in starch metabolism (the α-amylase VIT_03s0063g00410, the β-amylase, VIT_05s0051g00010, the isoamylase VIT_00s0131g00420 and the granule-bound starch synthase VIT_16s0022g00740), and four involved in the production of osmolytes such as trehalose (VIT_00s0233g00030, VIT_14s0036g01210, VIT_00s0173g00110 and VIT_11s0037g00710).

Microarray analysis also revealed that two CT highly modulated malate dehydrogenase (*MDH*) isogenes in two different cellular compartments showed opposite expression profiles during ripening, i.e. downregulation of the glyoxysomal isoform compared to control berries (VIT_03s0088g01190) but upregulation of the cytosolic isoform (VIT_07s0005g03360). Interestingly, another cytosolic *MDH *(VIT_07s0005g03350) was upregulated at EV and thereafter in CT berries but not expressed in controls, along with five NADP-dependent malic enzyme (*ME*) isogenes (VIT_03s0038g00040, VIT_16s0039g01050, VIT_16s0039g00580, VIT_15s0045g00190, VIT_04s0008g00180) and one phosphoenolpyruvate carboxykinase (VIT_07s0205g00070). Altogether these data suggest that the malate degradation is specifically influenced by cluster thinning, a hypothesis supported by the lower titratable acidity of CT berries compared to controls during ripening (Figure 1A and Table 1).

The oxidative burst that occurs in berries at the onset of ripening [22] seems to be enhanced by the thinning treatment, as supported by the upregulation of two alcohol dehydrogenases (*ADHs*, VIT_04s0044g01130 and VIT_04s0044g01120), three aldehyde dehydrogenases (*ALDHs*, VIT_01s0137g00090, VIT_01s0137g00080 and VIT_06s0004g02060) and two aldo/keto reductases (VIT_05s0062g00990 and VIT_08s0070g01010).

#### Genes belonging to the "Secondary Metabolism Process" category

We identified 108 CT highly modulated genes putatively involved in secondary metabolism (Table 6), 25 of which were downregulated (clusters 1-4), and 83 (including 70 not expressed in control berries) of which were upregulated (clusters 5-8). The downregulated genes included those related to the general phenylpropanoid pathway and phenolic acid metabolism. In particular, we detected two isoforms of 4-coumarate-CoA ligase (*4CLs*, VIT_17s0000g01790 and VIT_14s0171g00300), one caffeic acid 3-O-methyltransferase (*COMT*, VIT_08s0007g04520), one cinnamoyl-CoA reductase (VIT_16s0039g01670) and one cinnamyl alchohol dehydrogenase (VIT_19s0014g04980). A small number of genes involved in the flavonoid pathway were also downregulated in CT berries, including a flavonoid 3'-hydroxylase (*F3'H*, VIT_09s0002g01090), a *F3H *(VIT_16s0098g00860), a *LDOX *(VIT_08s0105g00380) and the leucoanthocyanidin reductase *LAR1 *(VIT_01s0011g02960). The downregulation of such genes may reflect the more robust suppression of proanthocyanidin biosynthesis in CT berries compared to controls, which may also account for the downregulation of a putative UDP-glucose:flavonoid glucosyltrasferase (VIT_16s0115g00340). This gene could be related to the reduced glucosylation of flavonoid compounds such as proanthocyanidin monomers produced during the herbaceous berry growth phase. Glucosylated proanthocyanidin does not accumulate in grapevine tissues but transient glucosylation might be necessary for the vacuolar import of monomers as reported in *Medicago truncatula *[23]. The downregulation of a geranylgeranyl diphosphatase synthase (VIT_15s0024g00850) and a geranylgeranyl reductase (VIT_17s0000g06280) in CT berries suggests that terpenoid metabolism is also affected by the treatment.

**Table 6:** CT highly modulated genes in the "Secondary metabolic process" functional category.

Gene_ID	Description	Metabolic Process	Profile	Expression in C	FC EV/BV	FC H/BV
VIT_02s0025g04020	S-N-methylcoclaurine 3'-hydroxylase	Alkaloid biosynthesis	1	**E**	-5.2	-2.4
VIT_12s0035g01080	carotenoid isomerase 1, chloroplast precursor	Carotenoid biosynthesis	1	**E**	-5.9	-1.7
VIT_06s0080g00810	lycopene beta cyclase (LYC)	Carotenoid biosynthesis	1	**E**	-6.1	-1.1
VIT_17s0000g09610	CYP71D10	Electron transport	1	**E**	-16.9	-5.2
VIT_09s0002g01090	flavonoid 3-monooxygenase	Flavonoid metabolism	1	**E**	-6.4	-2.9
VIT_16s0115g00340	UDP-glucose:flavonoid glucosyltransferase	Flavonoid metabolism	1	**E**	-9.8	-5.2
VIT_08s0007g04520	caffeic acid 3-O-methyltransferase	Phenolic acid metabolism	1	**E**	-6.2	-1.7
VIT_17s0000g01790	4-coumarate-CoA ligase 2	Phenylpropanoid metabolism	1	**E**	-5.4	-1.2
VIT_15s0024g00850	Geranylgeranyl diphosphate synthase	Terpenoid metabolism	1	**E**	-5.9	-1.2
VIT_17s0000g06280	geranylgeranyl reductase	Terpenoid metabolism	1	**E**	-5.0	-2.2

VIT_15s0046g03570	Salutaridine reductase	Alkaloid biosynthesis	2	**E**	-2.5	-6.6
VIT_15s0021g01060	CYP72A1	Electron transport	2	**E**	-3.4	-7.5
VIT_18s0001g09650	CYP81E1	Electron transport	2	**E**	-1.8	-5.1
VIT_16s0098g00860	Flavanone 3-hydroxylase	Flavonoid metabolism	2	**E**	-3.0	-7.7
VIT_08s0105g00380	Leucoanthocyanidin dioxygenase	Flavonoid metabolism	2	**E**	-3.7	-8.7
VIT_16s0039g01670	cinnamoyl-CoA reductase	Phenolic acid metabolism	2	**E**	-2.9	-5.4
VIT_14s0171g00300	4-coumarate-CoA ligase	Phenylpropanoid metabolism	2	**E**	-1.8	-6.0
VIT_16s0039g00990	Glutathione S-transferase 8 GSTU8	Secondary metabolism/Oxidative stress	2	**E**	-1.5	-5.1

VIT_19s0015g02500	CYP72A1	Electron transport	3	**E**	-1.2	-7.0
VIT_19s0014g04980	Cinnamyl alcohol dehydrogenase	Phenolic acid metabolism	3	**E**	-1.6	-6.9

VIT_07s0031g00620	zeaxanthin epoxidase (ZEP) (ABA1)	Carotenoids biosynthesis	4	**E**	-13.8	-9.0
VIT_03s0063g01590	CYP82C2	Electron transport	4	**E**	-6.8	-3.6
VIT_01s0011g02960	Leucoanthocyanidin reductase 1	Flavonoid metabolism	4	**E**	-4.6	-6.5
VIT_05s0049g01120	Glutathione S-transferase 25 GSTU7	Secondary metabolism/Oxidative stress	4	**E**	-5.2	-6.4
VIT_04s0023g01640	steroid 22-alpha-hydroxylase (CYP90B1) (DWF4)	Electron transport	4	**NE**	-10.0	-13.1

VIT_12s0134g00620	UDP-glucose:flavonoid glucosyltransferase	Flavonoid metabolism	5	**E**	10.3	0.5
VIT_06s0004g06400	UDP-glucose:flavonoid glucosyltransferase	Flavonoid metabolism	5	**E**	9.3	1.2
VIT_19s0015g02950	Secologanin synthase CYP72A1	Alkaloid biosynthesis	5	**NE**	7.4	1.4
VIT_04s0210g00030	strictosidine synthase	Alkaloid biosynthesis	5	**NE**	7.0	1.4
VIT_15s0048g01970	CYP708A1	Electron transport	5	**NE**	9.3	0.8
VIT_19s0027g00040	CYP72A59	Electron transport	5	**NE**	5.7	1.8
VIT_02s0012g02340	CYP76C4	Electron transport	5	**NE**	5.9	1.1
VIT_07s0129g00810	CYP81E8	Electron transport	5	**NE**	5.7	1.5
VIT_19s0090g01620	CYP89H3	Electron transport	5	**NE**	5.8	0.5
VIT_11s0016g01040	CYP92A2v4	Electron transport	5	**NE**	18.3	2.0
VIT_06s0009g02910	Flavonoid 3',5'-hydroxylase	Flavonoid metabolism	5	**NE**	18.7	1.7
VIT_06s0009g03010	Flavonoid 3',5'-hydroxylase [Vitis vinifera]	Flavonoid metabolism	5	**NE**	6.3	1.2
VIT_03s0110g00340	Cinnamyl alcohol dehydrogenase	Phenolic acid metabolism	5	**NE**	15.2	1.3
VIT_11s0016g01050	ferulate 5-hydroxylase	Phenolic acid metabolism	5	**NE**	8.2	0.7
VIT_08s0007g05050	4-coumarate--CoA ligase	Phenylpropanoid metabolism	5	**NE**	5.4	0.5
VIT_00s0240g00020	Glutathione S-transferase 23 GSTU23	Secondary metabolism/Oxidative stress	5	**NE**	21.1	2.7
VIT_01s0026g01370	glutathione S-transferase 29 GSTU18	Secondary metabolism/Oxidative stress	5	**NE**	5.8	0.7
VIT_19s0093g00290	GLUTATHIONE S-TRANSFERASE TAU 25	Secondary metabolism/Oxidative stress	5	**NE**	39.3	2.2
VIT_19s0014g02550	(-)-germacrene D synthase	Sesquiterpene biosynthesis	5	**NE**	5.5	0.6

VIT_16s0050g01590	UDP-glucose:flavonoid glucosyltransferase	Flavonoid metabolism	6	**E**	3.5	6.0
VIT_16s0039g01100	phenylalanine ammonia-lyase [Vitis vinifera]	Phenylpropanoid metabolism	6	**E**	2.6	7.0
VIT_16s0039g01240	phenylalanine ammonia-lyase [Vitis vinifera]	Phenylpropanoid metabolism	6	**E**	2.7	6.5
VIT_17s0000g09550	CYP71A26	Electron transport	6	**NE**	3.1	9.3
VIT_06s0009g03130	CYP79A2	Electron transport	6	**NE**	3.9	10.9
VIT_13s0106g00280	CYP79A2	Electron transport	6	**NE**	2.6	23.9
VIT_15s0048g01480	geraniol 10-hydroxylase	Monoterpenoids biosynthesis	6	**NE**	3.0	12.7
VIT_08s0040g03040	Glutathione S-transferase GSTL1	Secondary metabolism/Oxidative stress	6	**NE**	4.9	7.5
VIT_12s0034g01650	glutathione S-transferase Z2 GSTZ2	Secondary metabolism/Oxidative stress	6	**NE**	4.2	7.6
VIT_16s0100g01120	Stilbene synthase	Stilbene metabolism	6	**NE**	6.2	9.7
VIT_16s0100g01010	Stilbene synthase	Stilbene metabolism	6	**NE**	20.7	19.9
VIT_16s0100g00830	Stilbene synthase	Stilbene metabolism	6	**NE**	3.3	5.7
VIT_16s0100g00750	Stilbene synthase	Stilbene metabolism	6	**NE**	11.8	12.6
VIT_10s0042g00920	Stilbene synthase	Stilbene metabolism	6	**NE**	15.3	19.3
VIT_16s0100g00780	Stilbene synthase	Stilbene metabolism	6	**NE**	13.7	25.1
VIT_16s0100g01040	stilbene synthase - grape	Stilbene metabolism	6	**NE**	6.2	8.3
VIT_16s0100g00910	stilbene synthase - grape	Stilbene metabolism	6	**NE**	8.2	15.3
VIT_16s0100g01020	stilbene synthase [Vitis pseudoreticulata]	Stilbene metabolism	6	**NE**	9.8	15.4
VIT_16s0100g00960	stilbene synthase [Vitis pseudoreticulata]	Stilbene metabolism	6	**NE**	9.7	15.0
VIT_10s0042g00840	stilbene synthase [Vitis pseudoreticulata]	Stilbene metabolism	6	**NE**	25.6	38.3
VIT_16s0100g01030	stilbene synthase [Vitis quinquangularis]	Stilbene metabolism	6	**NE**	7.5	16.4
VIT_16s0100g01160	stilbene synthase [Vitis vinifera]	Stilbene metabolism	6	**NE**	9.2	13.4
VIT_16s0100g00810	stilbene synthase [Vitis vinifera]	Stilbene metabolism	6	**NE**	12.5	12.7
VIT_16s0100g01170	stilbene synthase 1 [Vitis vinifera]	Stilbene metabolism	6	**NE**	6.4	6.8
VIT_16s0100g00930	Stilbene synthase 2	Stilbene metabolism	6	**NE**	10.3	11.5
VIT_16s0100g00760	stilbene synthase 2 [Vitis sp. cv. 'Norton']	Stilbene metabolism	6	**NE**	20.4	25.5

VIT_01s0137g00560	CYP71B34	Electron transport	7	**E**	1.0	5.1
VIT_18s0001g12800	Dihydroflavonol 4-reductase	Flavonoid metabolism	7	**E**	1.0	5.9
VIT_17s0000g07210	flavonoid 3'-hydroxylase [Vitis vinifera]	Flavonoid metabolism	7	**E**	1.7	7.4
VIT_16s0039g01280	phenylalanine ammonia-lyase [Vitis vinifera]	Phenylpropanoid metabolism	7	**E**	2.3	8.7
VIT_12s0028g00920	Glutathione S-transferase 9 GSTF9	Secondary metabolism/Oxidative stress	7	**E**	1.3	5.6
VIT_19s0090g00140	5-alpha-taxadienol-10-beta-hydroxylase	Diterpenoid biosynthesis	7	**NE**	1.8	10.1
VIT_03s0091g00040	limonoid UDP-glucosyltransferase	Limonoids metablolism	7	**NE**	1.3	5.6
VIT_16s0039g01130	phenylalanine ammonia-lyase [Vitis vinifera]	Phenylpropanoid metabolism	7	**NE**	4.6	24.4
VIT_16s0100g01200	stilbene synthase	Stilbene metabolism	7	**NE**	3.6	12.9

VIT_19s0015g02700	Glutathione S-transferase 25 GSTU25	Secondary metabolism/Oxidative stress	8	**E**	9.9	4.1
VIT_19s0015g02890	Glutathione S-transferase 25 GSTU25	Secondary metabolism/Oxidative stress	8	**E**	10.8	7.0
VIT_19s0093g00150	Glutathione S-transferase 25 GSTU25	Secondary metabolism/Oxidative stress	8	**E**	8.7	5.8
VIT_13s0064g00810	9,10[9',10']carotenoid cleavage dioxygenase [Vitis vinifera]	Carotenoid metabolism	8	**NE**	10.0	7.2
VIT_19s0015g02100	CYP72A59	Electron transport	8	**NE**	27.5	6.2
VIT_06s0009g02850	CYP79A2	Electron transport	8	**NE**	6.2	2.0
VIT_07s0129g00770	CYP81D2	Electron transport	8	**NE**	61.2	8.7
VIT_07s0129g00830	CYP81D2	Electron transport	8	**NE**	15.4	6.0
VIT_03s0038g04620	isoflavone reductase	Isoflavone metabolism	8	**NE**	14.6	4.7
VIT_03s0038g04680	isoflavone reductase Bet v 6.0101	Isoflavone metabolism	8	**NE**	10.0	6.9
VIT_03s0038g04690	Isoflavone reductase protein 6	Isoflavone metabolism	8	**NE**	11.5	8.6
VIT_03s0038g04630	isoflavone reductase related protein	Isoflavone metabolism	8	**NE**	134.7	14.6
VIT_02s0025g02940	Caffeic acid O-3-methyltransferase	Phenolic acid metabolism	8	**NE**	6.2	3.4
VIT_15s0107g00210	Cinnamyl alcohol dehydrogenase	Phenolic acid metabolism	8	**NE**	15.1	4.2
VIT_13s0067g00560	Cinnamyl alcohol dehydrogenase	Phenolic acid metabolism	8	**NE**	26.8	12.4
VIT_02s0025g03110	Cinnamyl alcohol dehydrogenase	Phenolic acid metabolism	8	**NE**	10.2	7.6
VIT_13s0064g00290	Cinnamyl alcohol dehydrogenase	Phenolic acid metabolism	8	**NE**	11.1	4.9
VIT_19s0093g00110	Glutathione S-transferase 22 GSTU22	Secondary metabolism/Oxidative stress	8	**NE**	101.3	29.5
VIT_19s0015g02610	Glutathione S-transferase 25 GSTU25	Secondary metabolism/Oxidative stress	8	**NE**	36.3	8.6
VIT_19s0015g02730	Glutathione S-transferase 25 GSTU25	Secondary metabolism/Oxidative stress	8	**NE**	13.7	3.5
VIT_19s0027g00460	Glutathione S-transferase 25 GSTU25	Secondary metabolism/Oxidative stress	8	**NE**	25.0	8.1
VIT_06s0004g05670	Glutathione S-transferase 25 GSTU7	Secondary metabolism/Oxidative stress	8	**NE**	10.4	2.5
VIT_00s0240g00050	Glutathione S-transferase 8 GSTU19	Secondary metabolism/Oxidative stress	8	**NE**	116.7	16.8
VIT_19s0093g00160	Glutathione S-transferase 8 GSTU19	Secondary metabolism/Oxidative stress	8	**NE**	11.9	3.5
VIT_19s0093g00220	Glutathione S-transferase 8 GSTU19	Secondary metabolism/Oxidative stress	8	**NE**	51.5	8.2
VIT_19s0093g00310	Glutathione S-transferase 8 GSTU19	Secondary metabolism/Oxidative stress	8	**NE**	10.7	6.4
VIT_19s0015g02560	GLUTATHIONE S-TRANSFERASE TAU 25	Secondary metabolism/Oxidative stress	8	**NE**	28.1	3.6
VIT_12s0035g02100	Glutathione S-transferase Z1 GSTZ1	Secondary metabolism/Oxidative stress	8	**NE**	12.8	3.6
VIT_16s0100g00920	stilbene synthase - grape	Stilbene metabolism	8	**NE**	12.1	7.2

For each gene (Gene_ID) the annotation (Description), the function (Metabolic Process), the cluster number (Profile), the expression in C (E = expressed but not highly modulated; NE = not expressed) and the Fold Change (FC) referred to BV are reported.

The upregulated genes included several transcripts related to the general phenylpropanoid pathway, e.g. four *PAL *isogenes (VIT_16s0039g01100, VIT_16s0039g01240, VIT_16s0039g01280 and VIT_16s0039g01130) and one *4CL *(VIT_08s0007g05050). This suggests more phenolic precursors enter the multibranched phenylpropanoid pathway in CT berries. Indeed, many downstream pathways appeared to be specifically activated after cluster thinning. The induction of stilbene synthesis is suggested by the high upregulation of 19 *STSs *that are not expressed in control berries (many induced as early as the EV stage), the induction of isoflavone synthesis is indicated by the upregulation of four isoflavone reductase genes (VIT_03s0038g04620, VIT_03s0038g04680, VIT_03s0038g04690 and VIT_03s0038g04630), and the activation of phenolic acid metabolism is indicated by the upregulation of a *COMT *(VIT_02s0025g02940), a ferulate 5-hydroxylase (VIT_11s0016g01050) and five cinnamyl alcohol dehydrogenase genes (VIT_03s0110g0034, VIT_15s0107g00210, VIT_13s0067g00560, VIT_02s0025g03110 and VIT_13s0064g00290).

A small number of genes from the flavonoid pathway were also induced by cluster thinning, including the flavonoid 3',5'-hydroxylase genes *F3'5'Hi *(VIT_06s0009g02910), *F3'5'Hk *(VIT_06s0009g03010), the F3'Hb (VIT_17s0000g07210) [24] and the DFR (VIT_18s0001g12800) described by [25]. The expression profile of *F3'5'Hi *was further confirmed by real time RT-PCR (Additional File 3). Three putative UDP-glucose:flavonoid glucosyltrasferase transcripts (VIT_12s0134g00620, VIT_06s0004g06400 and VIT_16s0050g01590) were also upregulated, and these could also be involved in anthocyanin synthesis although this has yet to be confirmed.

In addition to the cinnamyl alcohol dehydrogenase genes, a few other genes responsible for the synthesis of flavor compounds were also induced by cluster thinning, including germacrene D-synthase (VIT_19s0014g02550), geraniol 10-hydroxylase (VIT_15s0048g01480), limonoid UDP-glucosyltrasferase (VIT_03s0091g00040) and a carotenoid cleavage dioxygenase (VIT_13s0064g00810), suggesting that the aromatic profile of berries is also affected by the treatment.

A very important consequence of cluster thinning was the high modulation of several members of the *GST *gene family (Table 6). Twenty different *GST *genes were activated in CT berries, including 16 tau-type (U), one phi-type (F), one lambda-type (L) and two zeta-type (Z) enzymes, according to the classification devised by Edwards et al. [26]. Five U-type and one F-type CT highly modulated *GSTs *were expressed but not highly modulated in control berries, whereas 13 U-type *GSTs *were not expressed in control berries at all. Only one of these transcripts (VIT_19s0015g02610) has already been functionally characterized in grapevine and it corresponds to *VvGST5 *(VIT_19s0015g02610 [21]). This was strongly upregulated between the EV and H stages in CT berries, which parallels the accumulation of anthocyanins. However, *VvGST5 *could not induce anthocyanin accumulation in transient assays carried out by Conn et al. [21], indicating that the enzyme is unlikely to have a direct role in anthocyanin biosynthesis. Only two *GST *transcripts (VIT_16s0039g00990 and VIT_05s0049g01120) were highly downregulated in CT berries.

#### Genes belonging to the "Transport" category

We identified 175 CT highly modulated genes in the "Transport" category, 36 of which are putatively involved in the transport of carbohydrates (10) or secondary metabolites (26) (Table 7). Of the ten putative carbohydrate transporters, seven were expressed but not highly modulated during ripening in control berries but were highly downregulated after cluster thinning. These included the sucrose transporter *VvSUC11 *(VIT_18s0001g08220 [27]), the polyol transporter *VvPMT5 *(VIT_03s0063g02250 [28]), an ERD6-like sugar transporter (VIT_07s0104g00830 [29]), a glucose-6-phosphate/phosphate-translocator required for import into plastids (VIT_11s0052g00430 [30]) and a succinate/fumarate mitochondrial transporter (VIT_08s0217g00010), all of which were downregulated at the EV stage. The remaining two transcripts, representing sucrose transporter *VvSUC27 *(VIT_18s0076g00250 [27]) and a 2-oxoglutarate/malate carrier protein (VIT_18s0001g07320) were downregulated at both the EV and H stages. In contrast, three dicarboxylate/tricarboxylate carriers (VIT_00s0188g00090, VIT_07s0031g02470 and VIT_08s0007g07270) were strongly upregulated by cluster thinning. Together with the strong induction of malate-degrading enzymes, the induction of dicarboxylate/tricarboxylate carriers reinforces the idea that thinning has a direct and specific impact on malate metabolism.

**Table 7:** CT highly modulated genes in "Transport" functional category specifically involved in carbohydrate and secondary metabolite translocation.

Gene_ID	Description	Metabolic Process	Profile	Expression in C	FC EV/BV	FC H/BV
VIT_11s0052g00430	Glucose-6-phosphate/phosphate-translocator	Carbohydrate transport	1	**E**	-6.0	-1.9
VIT_08s0217g00010	Succinate/fumarate mitochondrial transporter	Carbohydrate transport	1	**E**	-5.30	-2.86
VIT_03s0063g02250	POLYOL TRANSPORTER 5 (VIT_PMT5)	Carbohydrate transport	1	**E**	-7.1	-1.2
VIT_07s0104g00830	Sugar transporter ERD6-like 7	Carbohydrate transport	1	**E**	-9.5	-0.6
VIT_18s0001g08220	SUCROSE TRANSPORTER 11 (VIT_SUC11)	Carbohydrate transport	1	**E**	-5.6	-1.4

VIT_14s0108g00430	ABC transporter B member 16	Secondary metabolite transport	2	**E**	-3.9	-7.3
VIT_11s0052g01560	MATE efflux family protein	Secondary metabolite transport	2	**E**	-1.5	-6.1
VIT_07s0031g02550	ABC transporter G member 14	Secondary metabolite transport	2	**NE**	-9.2	-19.2
VIT_08s0056g00780	MATE efflux family protein	Secondary metabolite transport	2	**NE**	-5.8	-12.5
VIT_12s0059g02180	MATE efflux family protein	Secondary metabolite transport	2	**NE**	-7.7	-12.0

VIT_18s0001g07320	2-oxoglutarate/malate carrier protein, Mitochondrial	Carbohydrate transport	4	**E**	-12.11	-7.35
VIT_18s0076g00250	SUCROSE TRANSPORTER 27 (VIT_SUC27)	Carbohydrate transport	4	**E**	-16.5	-17.2

VIT_07s0031g02470	dicarboxylate/tricarboxylate carrier (DTC)	Carbohydrate transport	5	**NE**	19.03	2.69
VIT_06s0061g01460	ABC transporter G member 22	Secondary metabolite transport	5	**NE**	15.8	2.0
VIT_07s0031g00750	MATE efflux family protein	Secondary metabolite transport	5	**NE**	8.6	0.9
VIT_11s0052g01540	MATE efflux family protein	Secondary metabolite transport	5	**NE**	14.5	1.8

VIT_07s0005g04680	ABC transporter C member 9	Secondary metabolite transport	6	**NE**	4.6	6.7
VIT_09s0002g05360	ABC transporter g family pleiotropic drug resistance 12 PDR12	Secondary metabolite transport	6	**NE**	5.2	16.0
VIT_09s0002g05370	ABC transporter g family pleiotropic drug resistance 12 PDR12	Secondary metabolite transport	6	**NE**	9.7	17.0
VIT_09s0002g05400	ABC transporter g family pleiotropic drug resistance 12 PDR12	Secondary metabolite transport	6	**NE**	9.5	15.4
VIT_09s0002g05410	ABC transporter g family pleiotropic drug resistance 12 PDR12	Secondary metabolite transport	6	**NE**	3.3	8.4
VIT_09s0002g05440	ABC transporter g family pleiotropic drug resistance 12 PDR12	Secondary metabolite transport	6	**NE**	4.0	10.6

VIT_18s0001g11760	MATE efflux family protein	Secondary metabolite transport	7	**E**	0.8	5.1
VIT_09s0002g05490	ABC transporter g family pleiotropic drug resistance 12 PDR12	Secondary metabolite transport	7	**NE**	1.8	15.6

VIT_09s0002g02440	ABC transporter C member 12	Secondary metabolite transport	8	**E**	5.8	3.3
VIT_00s0188g00090	dicarboxylate/tricarboxylate carrier (DTC)	Carbohydrate transport	8	**NE**	32.96	15.30
VIT_08s0007g07270	dicarboxylate/tricarboxylate carrier (DTC)	Carbohydrate transport	8	**NE**	5.95	3.07
VIT_19s0015g00010	ABC transporter C member 9	Secondary metabolite transport	8	**NE**	9.9	8.5
VIT_19s0015g00040	ABC transporter C member 9	Secondary metabolite transport	8	**NE**	8.0	6.9
VIT_13s0101g00010	ABC transporter F member 2	Secondary metabolite transport	8	**NE**	56.9	23.4
VIT_03s0180g00300	ABC transporter F member 2	Secondary metabolite transport	8	**NE**	23.2	7.0
VIT_15s0024g00840	ABC transporter F member 2	Secondary metabolite transport	8	**NE**	55.7	17.2
VIT_09s0002g05430	ABC transporter g family pleiotropic drug resistance 12 PDR12	Secondary metabolite transport	8	**NE**	5.9	4.4
VIT_14s0068g01740	ABCNAP14	Secondary metabolite transport	8	**NE**	12.5	7.1
VIT_13s0019g05200	MATE efflux family protein	Secondary metabolite transport	8	**NE**	6.8	6.2
VIT_10s0042g00310	MRP-like ABC transporter	Secondary metabolite transport	8	**NE**	23.2	13.4

For each gene (Gene_ID) the annotation (Description), the function (Metabolic Process), the cluster number (Profile), the expression in C (E = expressed but not highly modulated; NE = not expressed) and the Fold Change (FC) referred to BV are reported.

Among the 26 putative secondary metabolite transporters highly modulated in CT berries, 19 belonged to the ATP-Binding Cassette (ABC) transporter family and seven to the Multidrug and Toxic Compound Extrusion (MATE) transporter family. Five of the transcripts were downregulated in CT berries, while 21 were upregulated (19 of which were not expressed in control berries at all). The ABC and MATE transporters were analyzed by querying the protein sequence of the probe consensus against the NCBI databases using BLASTX (Additional File 5). This was necessary because ABC and MATE transporters play multiple roles at the cellular level. None of the sequences we identified matched the grapevine anthocyanin-acylglucoside MATE transporters that have been functionally characterized [31]. Using *Arabidopsis thaliana *as a reference organism, we found that most of the transporter sequences matched significantly (e-value < 10^-50^) with an *Arabidopsis *protein homolog (Additional File 5). Furthermore, two CT highly upregulated MATE transporters matched TRANSPARENT TESTA 12 (AtTT12), a MATE transporter associated with flavonoid sequestration in vacuoles [32]. These sequences were VIT_18s0001g11760 (e-value = 3 × 10^-96^), which was not highly modulated in control berries but was upregulated in CT berries in the EV and H stages, and VIT_11s0052g01540 (e-value = 5 × 10^-74^), which was not expressed in control berries but was upregulated at the EV stage in CT berries. Exploration of the latest release of the *Vitis vinifera *genome revealed that the two MATE transporters mentioned above are not the closest homologs of *AtTT12 *(data not shown). However, the potential role of these two *TT12*-like genes in flavonoid transport cannot be ruled out.

### Genes highly modulated in control berries

The third group of 120 genes was highly modulated in control berries (Table 4). Although, like the common and CT highly modulated genes, these grouped into eight clusters, more than half of them were assigned to clusters 6 and 7, showing that most C highly modulated genes were upregulated during at least one ripening phase. There were 41 downregulated genes (clusters 1-4), six of which were not expressed at all in CT berries and 35 of which were expressed but not highly modulated during ripening after the cluster thinning treatment. There were 79 upregulated genes (clusters 5-8), 20 of which were not expressed in CT berries and 59 of which were expressed, but not highly modulated.

Using the annotation criteria described above, 15 C highly modulated genes were assigned to the "Transport" category, only one of which was involved in sugar transport (Table 8). This was the putative hexose transporter *VvHT5 *(VIT_05s0020g03140), which was induced strongly between the EV and H stages. Six genes involved in carbohydrate metabolism were highly modulated in control berries. We identified an *ADH *(VIT_06s0004g04320), an L-idonate dehydrogenase (VIT_16s0100g00290) which is involved in tartaric acid biosynthesis [33], a galactinol synthase (VIT_14s0060g00760) and a trehalose 6-phosphate phosphatase (VIT_18s0001g05300), all of which were downregulated, and an aldo-keto reductase (VIT_05s0077g01300) and a 2-phosphoglycerate kinase (VIT_06s0061g00280) that were upregulated. It is interesting to note that different isoforms of all these enzymes except L-idonate dehydrogenase were also found in the list of CT highly modulated genes. The *ADH *was highly downregulated in control berries but not highly modulated after cluster thinning, indicating that ADH activity is needed in CT berries as already suggested by the upregulation of two *ADH *genes in thinned vines.

**Table 8:** C highly modulated genes in the "Carbohydrate metabolic process", "Secondary metabolic process" and "Transport" (carbohydrate and secondary metabolite) functional categories.

Gene_ID	Description	Metabolic Process	Profile	Expression in C	FC EV/BV	FC H/BV
VIT_16s0100g00290	L-idonate dehydrogenase	Carbohydrate acid metabolism	2	**E**	-2.1	-6.5
VIT_18s0001g12180	CYP721A1	Electron transport	2	**E**	-3.9	-11.2
VIT_14s0060g00760	galactinol synthase	Sugar metabolism	2	**E**	-2.3	-5.2

VIT_18s0001g11470	CyP82A3	Electron transport	3	**E**	-2.1	-6.6
VIT_06s0004g04320	alcohol dehydrogenase	Fermentative metabolism	3	**E**	-1.8	-8.7
VIT_18s0001g05300	trehalose-6-phosphate phosphatase	Starch and sucrose metabolism	3	**NE**	-1.1	-5.7

VIT_05s0077g01300	Aldo-keto reductase	Aldehyde detoxification pathways (oxidative stress)	6	**E**	5.1	5.0
VIT_10s0003g05450	reticuline oxidase precursor	Alkaloid biosynthesis	6	**E**	2.8	10.1
VIT_16s0039g00880	CYP89H3	Electron transport	6	**E**	7.3	18.0
VIT_18s0001g12690	Isoflavone reductase protein 4	Isoflavone metabolism	6	**E**	2.0	6.2

VIT_06s0061g00280	2-phosphoglycerate kinase	Glycolysis	7	**E**	1.2	6.0
VIT_11s0065g00350	trans-cinnamate 4-monooxygenase (C4H)	Phenolic acid metabolism	7	**E**	0.8	5.6
VIT_05s0020g03140	Hexose Transporter 5 (VvHT5)	Secondary metabolite transport	7	**E**	1.1	9.0
VIT_07s0151g01070	copalyl diphosphate synthase	Diterpenoid biosynthesis	7	**NE**	1.2	11.2
VIT_04s0023g01290	UDP-glucose:flavonoid glucosyltransferase	Flavonoid metabolism	7	**NE**	1.8	18.9

For each gene (Gene_ID) the annotation (Description), the function (Metabolic Process), the cluster number (Profile), the expression in C (E = expressed but not highly modulated; NE = not expressed) and the Fold Change (FC) relative to the BV stage are reported.

Very few genes related to phenol metabolism were highly modulated in control berries, the exceptions being an isoflavone reductase (VIT_18s0001g12690), a cinnamate-4-hydroxylase (VIT_11s0065g00350) and one transcript putatively involved in anthocyanin modification (VIT_04s0023g01290) which were upregulated. The third of these transcripts was not expressed at all in CT berries as confirmed by real-time RT-PCR (Additional File 3). We did not identify any *GST *genes that were highly modulated in control berries, or any genes with a putative role in secondary metabolite transport or storage.

### Cluster thinning mainly affects the berry transcriptome at the end of veraison

To complete the analysis of our microarray results, we compared the C and CT berry transcriptomes at each time point using a SAM unpaired comparison with a FDR of 2%. This revealed 4123 genes that were differentially expressed in C and CT berries at EV, and 178 at H, in each case with a fold change ≥ 2 (Additional File 6). No genes were found to be differentially expressed in C and CT berries at the BV stage. As anticipated by the previous PCA, these data indicate that C and CT berries at the BV stage are indistinguishable at the transcriptomic level, the main changes occurring at the EV stage, followed by minor further changes at the H stage. As described above, we then focused on genes with a fold change ≥ 5, narrowing the analysis to 1167 differentially-expressed genes at EV and 53 at H, 40 of which were common to both stages. We found that 833 genes were upregulated and 347 were downregulated in CT berries at EV and/or at H.

Approximately 80% (940) of these genes had already been identified as differentially expressed either in C or in CT berries by the cluster analysis based on SAM multiclass described above (Additional File 7). Because these genes were mined from the microarray data using two different approaches, we can have greater confidence that they represent a genuine molecular response to thinning and most (893) are indeed included in the list of genes highly modulated in response to CT. The remaining 20% of genes identified only by the direct comparison at each time point represent genes with a less than five-fold difference in expression between C and CT during ripening, and which were therefore excluded from the first cluster analysis.

As described for the first dataset, we determined the functional categories of the identified genes and focused on those related to carbohydrate metabolism, secondary metabolism and the transport of carbohydrates and secondary metabolites (Table 9). All genes belonging to these categories were differentially expressed in C and CT berries, but only at the EV stage. Only two genes were related to carbohydrate metabolism: a trehalose-phosphate synthase potentially involved in stress tolerance which was downregulated in CT berries (VIT_19s0014g00300), and a pyruvate decarboxylase involved in cellular respiration which was upregulated in CT berries (VIT_15s0024g00630). All the genes related to secondary metabolism were upregulated in CT berries, including two cinnamyl alcohol dehydrogenases (VIT_13s0047g00760 and VIT_00s0371g00010), two GSTs (VIT_07s0104g01810 and VIT_06s0004g05680) and two UDP-glucose:flavonoid 5,3-O-glucosyltransferases (VIT_18s0041g00840 and VIT_18s0041g00950). The induction of these genes reinforces our cluster analysis results, which already showed that the expression of several cinnamyl alcohol dehydrogenase, GST and putative UDP-glucose:flavonoid glucosyl transferase genes was triggered by thinning. The same holds true for the four secondary metabolite transporters, three of which were upregulated (VIT_15s0045g01030, VIT_14s0030g00900, VIT_10s0003g03470) and one of which was downregulated in CT berries (VIT_16s0100g00350).

**Table 9:** Differentially expressed genes in C and CT berries at the EV stage belonging to "Carbohydrate metabolic process", "Secondary metabolic process" and "Transport" functional categories.

Gene_ID	Description	Metabolic Process	FC CT/C at EV
VIT_19s0014g00300	alpha, alpha-trehalose-phosphate synthase	Stress tolerance	-5.20
VIT_15s0024g00630	pyruvate decarboxylase isozyme 2	Glycolysis	5.52
VIT_13s0047g00760	Cinnamyl alcohol dehydrogenase	Phenolic acid metabolism	6.58
VIT_00s0371g00010	cinnamyl alcohol dehydrogenase	Phenolic acid metabolism	5.03
VIT_19s0135g00230	CYP72A1	Electron transport	7.69
VIT_19s0015g02520	CYP72A1	Electron transport	5.28
VIT_19s0015g02540	CYP72A59	Electron transport	7.30
VIT_19s0015g02780	CYP72A59	Electron transport	5.48
VIT_07s0104g01810	Glutathione S-transferase 13 GSTF13	Secondary metabolism/Oxidative stress	8.03
VIT_06s0004g05680	Glutathione S-transferase 25 GSTU7	Secondary metabolism/Oxidative stress	5.11
VIT_18s0041g00840	UDP-glucose: flavonoid 5,3-O-glucosyltransferase	Flavonoid metabolism	10.10
VIT_18s0041g00950	UDP-glucose: flavonoid 5,3-O-glucosyltransferase	Flavonoid metabolism	5.04
VIT_16s0100g00350	ABC transporter B member 8	Secondary metabolite transport	-5.12
VIT_15s0045g01030	MRP-like ABC transporter MRP6	Secondary metabolite transport	9.44
VIT_14s0030g00900	MRP-like ABC transporter MRP6	Secondary metabolite transport	5.09
VIT_10s0003g03470	MRP5	Secondary metabolite transport	7.80

For each gene (Gene_ID) the annotation (Description), the function (Metabolic Process), and the Fold Change (FC) between CT and C at the EV stage are reported.

## Discussion

Although many previous studies have considered the impact of increasing the source/sink ratio on grape berry composition, ours is the first investigation to look at the consequences of cluster thinning on global gene expression profiles, which is the basis of most of the observed physiological and biochemical changes.

Although there is only limited evidence for a strict relationship between yield and quality [34], in this study the effectiveness of thinning reflected the suboptimal leaf area to yield ratio of control vines (0.6 m^2^/kg). Increasing this ratio to 1.2 m^2^/kg by cluster thinning boosted the levels of sugars and anthocyanins and reduced acidity at harvest. Several authors have reported similar effects on sugars and anthocyanins [35,36], but there is disagreement on the impact of thinning on titratable acidity, with reports suggesting that acidity is unaffected [7,37], slightly increased [1] or decreased by yield reduction [2,38]. In all cases, the effect on acidity seems to be related to the impact of cluster thinning on ripening, particularly the soluble solids content, because thinning reduces acidity only when the soluble solids concentration is strongly and positively affected ([38,39] and this study).

The grouping of gene expression profiles by principal component analysis showed that C and CT berries could be distinguished as early as two weeks after treatment. Microarray data were then analyzed by two different approaches. Genes that were differentially expressed in C and CT berries were initially clustered on the basis of their expression profiles, and then we carried out a direct comparison of C and CT transcriptomes at each time point.

The first approach revealed such a large number of genes modulated during ripening that we applied a > 5 fold change cut-off threshold before assigning a gene to the modulated group. This resulted in 2466 transcripts that were considered to be modulated in CT berries and 567 that were modulated in C berries, including 447 that were common to both treatments. Approximately half of the common genes were more strongly modulated in CT berries, including several downregulated genes involved in photosynthesis, carbon utilization, carbohydrate metabolism, cell wall modifications and hormone metabolic processes that are already known to have a role in berry ripening [22,40], and several upregulated genes involved in the normal ripening process (e.g. genes related to secondary metabolite biosynthesis). These data strongly suggest that the entire course of berry ripening is enhanced by the cluster thinning.

An interesting and unexpected result of the microarray analysis was the relatively large number of genes (2019) highly modulated only in CT berries. More than a half of these genes were never expressed in control berries at any ripening stage, and were activated uniquely by the cluster thinning treatment. This shows that thinning is able to trigger the transcription of genes that otherwise would not be activated in untreated berries and therefore that the effect of thinning goes beyond the simple enhancement or acceleration of the normal ripening process. This appears to affect many different metabolic and cellular processes because the CT highly modulated genes are distributed throughout all 18 functional categories we considered.

The CT high modulation of genes involved in carbohydrate metabolism supports the impact of cluster thinning on sugar accumulation commencing at the EV stage. This reflects the achievement of an optimal balance between leaf area and yield in thinned vines (1.2 m^2^/kg) compared to controls (0.6 m^2^/kg) as previously reported [2-7]. Interestingly, all CT highly modulated genes involved in sugar transport (including *VvSUC11 *and *VvSUC27*) were downregulated in CT berries but expressed at a constant level throughout ripening in controls. The downregulation of sucrose transporters in CT berries contrasts with the increase in sucrose transporter mRNA during berry development reported by Davies et al. [27] and the general enhancing effect of thinning on the entire ripening process. One possible explanation is that the higher sugar concentration triggers negative feedback that affects the sucrose transporters. Indeed the presence of sugar-response elements in the promoters of various sucrose transporter genes, potentially acting as *cis*-regulatory elements involved in sugar signaling, has recently been reported [41].

Despite the significant decline in starch concentration following veraison, several genes involved in starch biosynthesis and modification are modulated during ripening [40]. We detected both highly up- and down-regulated genes in CT involved in starch degradation, and their role is unclear given that developing CT berries accumulate large amounts of sugar. Simultaneous starch synthesis and degradation may facilitate the unloading and storage of sugars in the ripening fruit [39]. Starch-degrading enzymes might also provide carbon backbones for the biosynthesis of secondary metabolites, which could also act as signaling molecules in the regulation of genes controlling phenylpropanoid synthesis [17,42].

The strong induction of genes encoding malate-degrading enzymes in CT berries, together with the induction of dicarboxylate-tricarboxylate carriers (which transport malate across the mitochondrial membrane thus supplying substrates for the Krebs cycle) supports the specific modulation of malate metabolism by cluster thinning. Malate, whose catabolism is considered responsible of total acidity reduction in grape berry after véraison [43] is liberated from the vacuole in post-véraison becoming available for catabolism through various avenues [44,45] and, with the advance of ripening, malic acid is likely a vital source of carbon.

Although advanced malate degradation is heavily dependent upon the extent to which berry temperature is elevated [46,47] in response to increased sunlight exposure, this condition was not tested in our experiment as the cluster microclimate was not modified by the removal of one cluster of each shoot, maintaining an unchanged canopy structure.

In this respect, our microarray data suggest that malic acid catabolism is accelerated by cluster thinning following the general enhanced ripening process as seen in other researches [48].

Genes involved in the metabolism of phenylpropanoids and aromatic compounds were strongly modulated in CT berries. Several transcripts involved in phenolic acid, stilbene, flavonoid and isoflavonoid metabolism were affected by the treatment, suggesting that these distinct branches of the phenylpropanoid pathway are directly affected by the increased source/sink balance. However it is possible that the synthesis of phenolic compounds such as stilbenes or isoflavonoids could be part of a systemic response to wounding resulting from the removal of berry clusters, since these compounds are normally produced by the plant in response to stress conditions such as wounding or interactions with pathogens [49,50].

The anthocyanin content of CT berries was higher than that of controls (Figure 1B and Table 1) but the only CT highly upregulated transcript related to the flavonoid/anthocyanin pathway that could explain this result was *DFR*. However, looking specifically for known anthocyanin-related transcripts, we found that *VvGST4 *(VIT_04s0079g00690) and *VvMYBA1 *(VIT_02s0033g00410) were also more strongly upregulated in CT berries compared to controls (Additional File 2). The high induction of three flavonoid glucosyltransferases and the large number of CT highly upregulated *GSTs *and transporters of the ABC and MATE families may also play an important role in triggering anthocyanin biosynthesis, although their precise functions in berry ripening remain to be determined. Several putative flavonoid-related transcripts such as *F3H*, *LDOX*, *LAR1 *and UDP-glucose:flavonoid glucosyltransferase were highly downregulated in CT berries, which may reflect the slowing down of the synthesis of non-anthocyanin flavonoid compounds such as proanthocyanidin. Several reports indicate that agronomic treatments such as water stress and cluster thinning can increase total anthocyanins and also produce a shift in their profile [3,4,51,52]. The anthocyanin composition of CT berries differed from controls in our study, with higher relative levels of peonidin 3-glucoside and total 3'4'-OH anthocyanins, in agreement with earlier reports [3,51]. This might reflect the specific upregulation of *F3'Hb *in CT berries during the EV and H phases. Although *F3'5'Hi *and *F3'5'Hk *were upregulated in CT berries and not expressed at all in controls, there was a significant decrease in the levels of 3'4'5'-OH anthocyanins at harvest, probably because this expression pattern was restricted just to the EV stage. This suggests that the biosynthesis of 3'4'-OH and 3'4'5'-OH anthocyanins is controlled independently [5].

Cluster thinning is known to change the aromatic profile of grape berries and increase the resveratrol content in wine [53,54]. In agreement, our microarray data reveal the induction of many putative aroma-modifying transcripts (e.g. geraniol 10-hydroxylase, germacrene D-synthase, as well as two isoforms each of ADH and ALDH) and the specific upregulation of 19 *STSs*.

The number of genes highly modulated in control berries (567) was small in comparison with previous studies [22,40] but this reflects our stringent application of the requirement for a fold change > 5. Only 120 of the control highly modulated genes were not highly modulated in CT berries, none of which were involved in carbohydrate or secondary product metabolism. Therefore we assume they play a relatively minor role in the control of berry quality traits at harvest.

The direct comparison of C and CT transcriptomes at each time point indicated that the greatest number of genes differentially expressed in C and CT berries were found at the EV stage, whereas only minor differences were detectable at harvest. This generally supported the cluster analysis results because almost all the differentially expressed genes had already been detected. The novel data obtained by direct comparison related mainly to genes whose expression was only slightly modulated during ripening.

## Conclusions

We investigated the effect of cluster thinning on the Sangiovese berry transcriptome in association with agronomic parameters and the biochemical properties of berries during ripening. The increased source-sink ratio achieved by cluster thinning reflected the optimal leaf area/crop weight ratio in thinned vines (1.2 m^2^/kg) compared to 0.6 m^2^/kg in controls. This caused the sugar and anthocyanin content to increase from veraison to harvest at an accelerated rate, along with extensive transcriptomic reprogramming involving both the increased modulation of genes that are normally regulated during ripening and the induction of a large group of genes that are not expressed in ripening control berries. Cluster thinning therefore accelerates the normal ripening process but also superimposes a metabolic environment involving the induction of novel processes not found in ripening control berries and the repression of some pathways that are part of the normal ripening process. Although the possibility that the transcriptomic reprogramming was partially an effect and not only the cause of the observed metabolic changes cannot be excluded, our data provide a significant contribution to current understanding of the molecular consequences of cluster thinning, specifically the impact of increasing the source/sink ratio at veraison on carbohydrate and anthocyanin accumulation.

## Methods

### Plant material

The trial was carried out in 2008 on adult Sangiovese (*Vitis vinifera *L.) vines, clone 12T grafted to SO4, in a vineyard in Bologna, Italy (44°30'N, 11°24'E), with North-South oriented rows (vine spacing 1 m × 2.8 m, vertical shoot positioned spur pruned cordon training system (12 buds per vine), with cordon height at 1.0 m above ground and a canopy height of 1.3-1.4 m). Pest management was carried out according to Regione Emilia Romagna local practice. Shoots were manually trimmed when most started to outgrow the top wire, which occurred on Julian Day (JD) 192. Nine vines per treatment, with the same cluster number at flowering (16 per vine), were selected in a single uniform row and each vine was randomly assigned in three blocks to the two following groups: control (C, no treatment) and cluster thinning (CT, removal of 50% of total clusters per vine at veraison, JD 211).

### Berry sampling

Samples of 40 berries, taken from three vines in each block, were collected at the following stages: a) beginning of veraison (BV, berries softening and °Brix ~8; JD 211); b) full veraison (one week later; JD 219); c) end of veraison (EV, soft and fully-colored berries; JD 227); d) ripening (two weeks after stage c, JD 250); e) full ripening (H, harvest, JD 266). The samples were divided in two and 20 berries were processed immediately in order to monitor berry ripening, while the rest were frozen at -20°C in preparation for HPLC analysis of anthocyanins. We also collect 30 additional samples at stages a, c and e (same method as above), which were immediately frozen in liquid nitrogen and stored at -80°C for subsequent microarray analysis.

### Agronomic parameters and biochemical analysis

Final vine leaf area was estimated after the achievement of a linear relationship between shoot length (cm) and leaf area (cm^2^) for at least 15 shoots for both C and CT samples collected from vines at the end of shoot growth. Leaf area was measured with a leaf area meter (LI-3000A, Li-Cor Biosciences, Lincoln, Nebraska, USA), ensuring the contribution of the main and lateral leaf areas were kept separate. Two separate correlations for the main and lateral areas (y) and shoot lengths (x) were so established (C main: y = 16.212 x, R^2 ^= 0.70; C laterals: y = 14.834x, R^2 ^= 0.70; CT main: y = 13.441x, R^2 ^= 0.71; CT laterals: y = 15.844x, R^2 ^= 0.89). After leaf fall, each shoot per vine was then measured and the shoot lengths were used to estimate the corresponding pooled total leaf area per vine using the linear relationship calculated above.

The must obtained after crushing the first 20 berries per sample was immediately filtered through a strainer and a drop was used for °Brix analysis with a temperature-compensating CR50 refractometer (Maselli Misure Spa, PR, Italy). We then diluted 5 ml of the same must in seven volumes of double distilled water and titrated this against 1 N, 0.5 N or 0.25 N NaOH (Sigma-Aldrich, St. Louis, MO, USA) with a Crison Compact Tritator (Crison, Barcelona, Spain) according to the stage of berry ripening, to obtain pH and titratable acidity data (expressed as g/L of tartaric acid equivalents).

The second 20 berries were used to extract anthocyanins for HPLC analysis as described by Mattivi et al. [55], using a Waters 1525 instrument (Waters, Milford, MA) equipped with a diode array detector (DAD) and using a Phenomenex (Castel Maggiore, BO, Italy) reversed-phase column (RP18, 250 mm × 4 mm, 5 μM). Anthocyanins were quantified at 520 nm using an external calibration curve with malvidin-3-glucoside chloride as the standard (Sigma-Aldrich, St. Louis, MO).

We recorded cluster number and weight, berry weight and total yield per vine for each tested vine at harvest. Statistical analysis of agronomic parameters and biochemical data were carried out using the mixed General Linear Model (GLM) procedure of the SAS statistical package (SAS Institute, Cary, NC), and treatment comparisons were carried out using the Tukey test.

### Microarray analysis

Total RNA for microarray analysis was isolated from ~200 mg of the ground berry tissue without seeds using the Spectrum™ Plant Total RNA kit (Sigma-Aldrich, St. Louis, MO). RNA quality and quantity were determined using a Nanodrop 2000 instrument (Thermo Scientific, Wilmington, DE) and a Bioanalyzer Chip RNA 7500 series II (Agilent, Santa Clara, CA). The cDNA synthesis, labeling, hybridization and washing reactions were performed according to the NimbleGen Arrays User's Guide (V 3.2). Each hybridization was carried out on a NimbleGen microarray 090818 Vitis exp HX12 (Roche, NimbleGen Inc., Madison, WI), representing 29,549 predicted genes on the basis of the 12X grapevine V1 gene prediction version http://srs.ebi.ac.uk/. The chip probe design is available at the following URL: http://ddlab.sci.univr.it/FunctionalGenomics/.

The microarray was scanned using a ScanArray 4000XL (Perkin-Elmer) at 532 nm (Cy-3 absorption peak) and GenePix Pro7 software (Molecular Devices, Sunnyvale, CA, USA) according to the manufacturers' instructions. Images were analyzed using *NimbleScan v2.5 *software (Roche), which produces Pair Files containing the raw signal intensity data for each probe and Calls Files with normalized expression data derived from the average of the intensities of the four probes for each gene. The normalized gene expression data were finally converted to log_2 _values. All microarray expression data are available at GEO under the series entry GPL13936.

Pearson Correlation analysis and Principal Component Analysis (PCA; SIMCA P+ Umetrics, Umea, Sweden) were carried out to evaluate the robustness of the three biological replicates in each sample. A gene was considered to be expressed if the normalized expression value was higher than the value obtained by averaging the fluorescence of the negative control present on the chip, for at least two of the three biological replicates. A Significance Analysis of Microarrays (SAM) was implemented using TMeV software http://www.tm4.org/mev, with a false discovery rate of 2%. Cluster analysis was performed by the k-means method with Pearson's correlation distance (TMeV) comparing EV and H gene expression to BV.

### Real-time PCR

First strand cDNA was synthesized using 1 μg of total RNA as the template and the Improm-II™ Reverse Transcription system (Promega), according to the manufacturer's instructions. The total RNA was derived from the three biological replicates used for microarray analysis and all RNA samples were first treated with DNase I (Promega). Gene-specific primers were designed for six genes using the sequence information in the 3'-UTR, using actin and elongation factor 1 (EF1) genes as references [56] (Additional File 8). Primers and cDNA were mixed with the Power SYBR^® ^Green PCR Master Mix (Applied Biosystems, Foster City, CA, USA) and the reaction was carried out on an ABI PRISM StepOne Sequence Detection System (Applied Biosystems, Foster City, CA, USA) using the following cycling conditions: 50°C hold for 2 min and a 95°C hold for 10 min followed by 45 cycles at 95°C for 30 s, 55°C for 30 s and 72°C for 20 s. Nonspecific PCR products were identified by the dissociation curves. Amplification efficiency was calculated from raw data using LingRegPCR software [57]. The relative expression ratio was calculated for development time points relative to the first sampling time point (beginning of veraison, BV) according to the Pfaffl equation [58]. Standard error (SE) values were calculated according to Pfaffl et al. [59]. The final data was calculated as previously reported [60].

## Authors' contributions

CP set up the RNA extraction, performed microarray and HPLC experiments, participated in data analysis, prepared figures and tables and wrote the initial manuscript draft. SZ set up the real-time RT-PCR protocol, performed data analysis and participated in the drafting of the manuscript. GBT participated in the interpretation of data and supervised and critically revised the manuscript. GA and GV sampled the material and performed the HPLC analysis. SDS set up the microarray experiment and participated in the real time RT-PCR experiments and gene annotation. CI conceived the study and participated in the project design. MP and IF conceived the study, participated in its design and coordination and contributed to and critically revised the manuscript. It is the authors' opinion that CP and SZ contributed equally as first authors to this manuscript. All authors have read and approved the final version of the manuscript.

## Supplementary Material

Additional file 1 **Differentially expressed genes along berry development displaying a two-fold or greater change in transcript abundance between EV and BV or EV and H, in CT and C treatments**. For each gene the annotation, the EV/BV and the H/BV Fold-change (FC) are indicated. Data obtained for CT and C are listed in two separate worksheets. Click here for file

Additional file 2 **Differentially expressed genes along berry development displaying a five-fold or greater change in transcript abundance between EV and BV or EV and H, in CT and C treatments**. For each gene the annotation, the gene ontology, the EV/BV and the H/BV Fold-change (FC), the expression profile and the expression behavior in the other treatment are reported. Data obtained for CT and C are listed in two separate worksheets. Click here for file

Additional file 3 **Real time RT-PCR validation of six selected genes. Expression profiles measured by real time RT-PCR are determined by calculating the relative expression ratio value for each stage relative to the BV stage**. Real time RT-PCR data are reported as means ± SE of three biological replicates, obtained by using two reference genes. An actin beta/gamma 1 (VIT_12s0178g00200) and an elongation factor 1 (VIT_06s0004g03220) were used as control genes. For each gene the expression profile obtained by microarray analysis is shown on the right side. A-B: Vacuolar invertase 1, GIN 1 (VIT_16s0022g00670); C-D: PAL [Vitis vinifera] (VIT_16s0039g01120); E-F: Flavonol synthase (VIT_18s0001g03430); G-H: β-Fructosidase -invertase (VIT_00s2527g00010); I-J: Flavonoid 3’5’-hydroxylase (VIT_06s0009g02910); K-L: UDP-glucose:flavonoid glucosyltransferase (VIT_04s0023g01290). Click here for file

Additional file 4 ** Differentially expressed genes along berry development displaying a similar expression profile between C and CT**. For each gene, the annotation, the gene ontology, the profile and the CT/C Fold-change (FC) ratio at EV and at H are reported. Click here for file

Additional file 5 **Differentially expressed genes along CT berry development encoding ABC and MATE transporters**. Analysis was performed by querying the protein sequence of the probe consensus against the NCBI databases using BLASTX. For each gene the correspondent Arabidopsis thaliana homologue, the NCBI reference sequence and E-value are specified. Click here for file

Additional file 6 **Differentially expressed genes displaying a two-fold or greater change in transcript abundance between C and CT at EV and H time points**. For each gene the description and the CT/C Fold-change (FC) are indicated. Data obtained for EV and H are listed in two separate worksheets. Click here for file

Additional file 7 **Differentially expressed genes displaying a five-fold or greater change in transcript abundance between C and CT at EV and /or H**. For each gene the CT/C Fold-change (FC) at EV and /or H, the description, the gene ontology, the presence of the gene among genes identified by cluster analysis as CT specific, C specific, common to C and CT with a different expression profile and common to C and CT with the same expression profile, are reported.. Click here for file

Additional file 8 **List of the primers used for real time RT-PCR validation experiment**. Click here for file
