# Supplementary figures and images for: Increasing the source/sink ratio in Vitis vinifera (cv Sangiovese) induces extensive transcriptome reprogramming and modifies berry ripening
Source: BMC Genomics. 2011 Dec 23;12:631. doi: 10.1186/1471-2164-12-631 (PMC3283566; doi:10.1186/1471-2164-12-631)

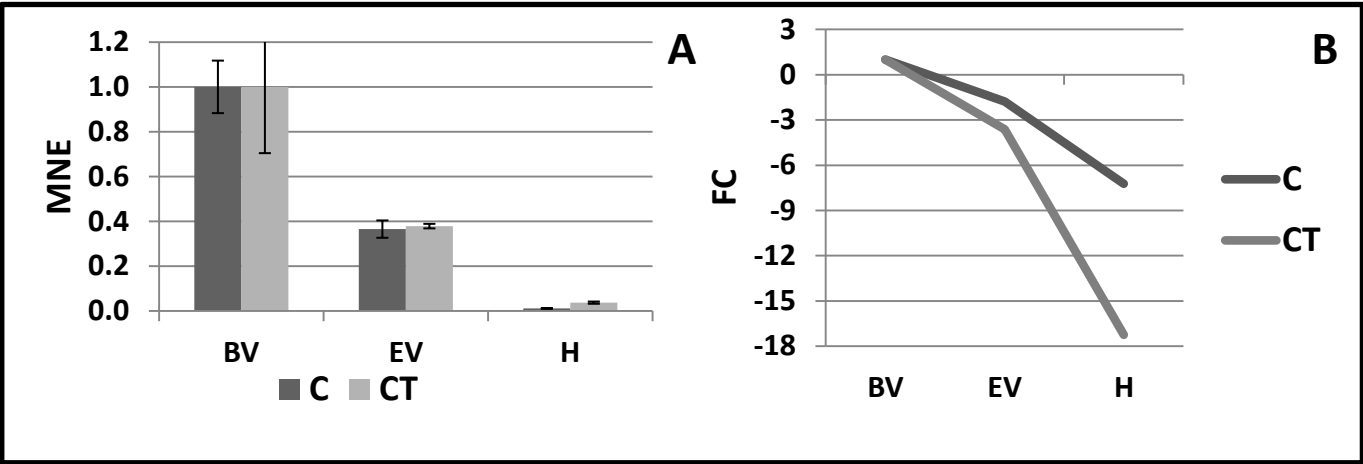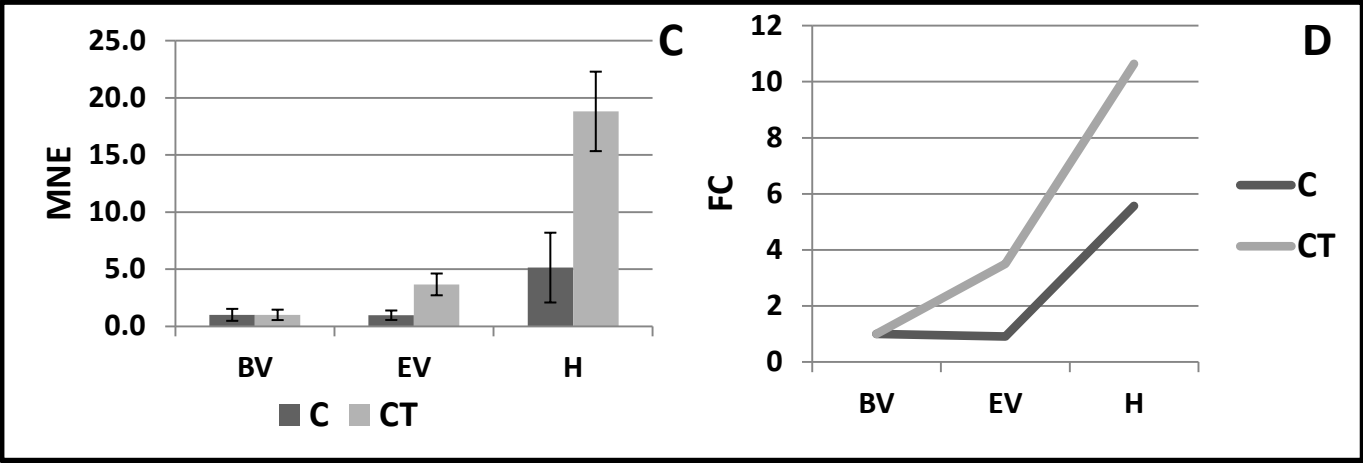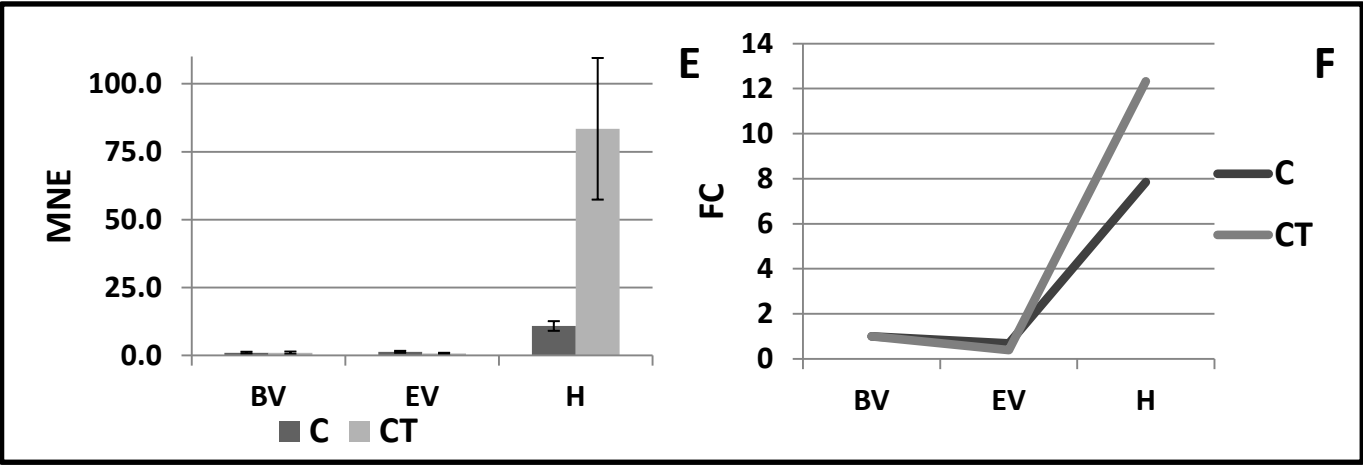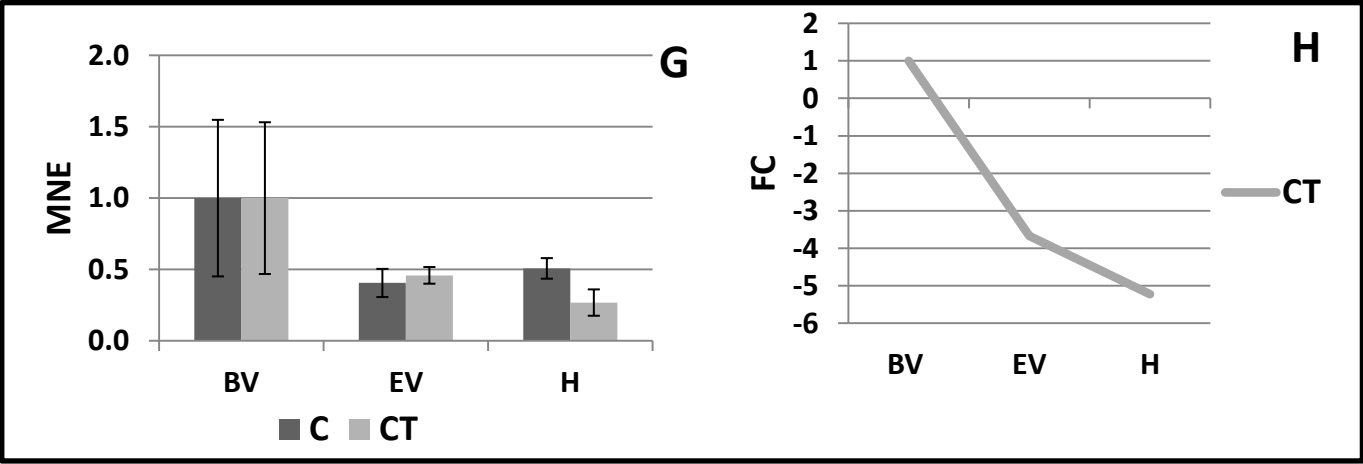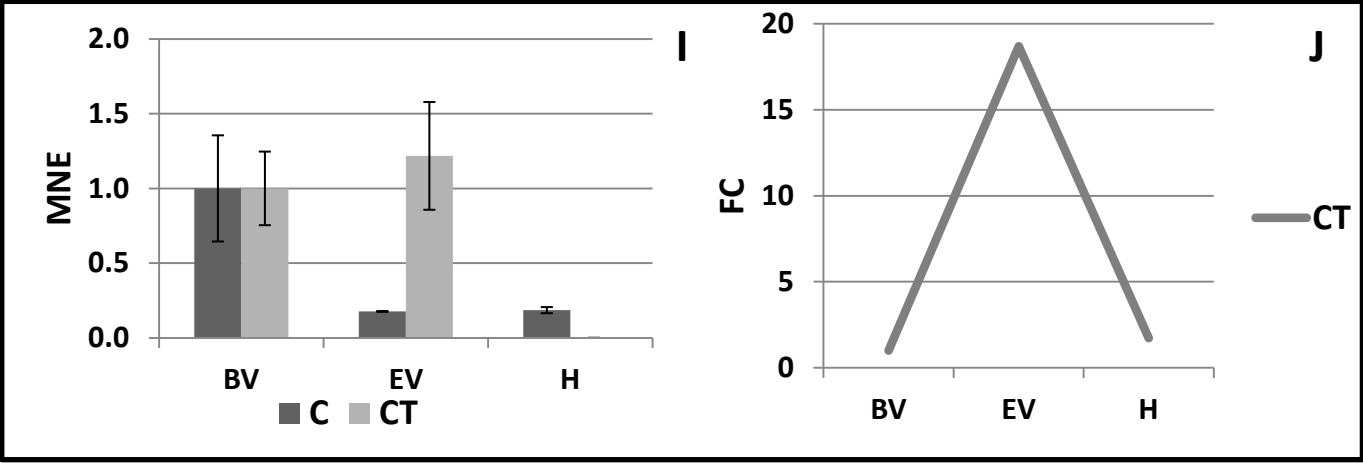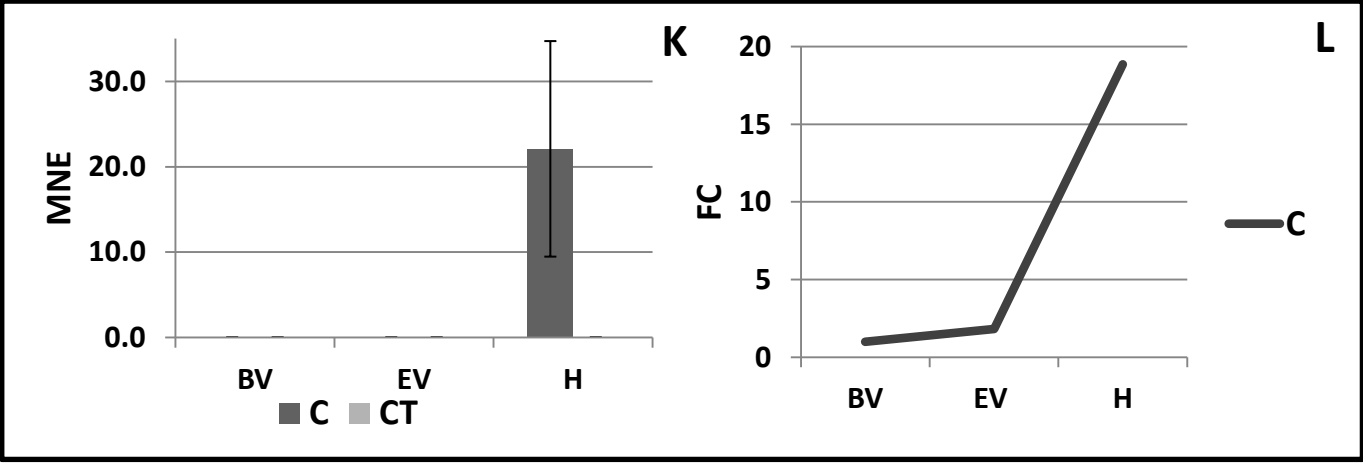

Supplement: Additional file 3 — Real time RT-PCR validation of six selected genes. Expression profiles measured by real time RT-PCR are determined by calculating the relative expression ratio value for each stage relative to the BV stage. Real time RT-PCR data are reported as means ± SE of three biological replicates, obtained by using two reference genes. An actin beta/gamma 1 (VIT_12s0178g00200) and an elongation factor 1 (VIT_06s0004g03220) were used as control genes. For each gene the expression profile obtained by microarray analysis is shown on the right side. A-B: Vacuolar invertase 1, GIN 1 (VIT_16s0022g00670); C-D: PAL [Vitis vinifera] (VIT_16s0039g01120); E-F: Flavonol synthase (VIT_18s0001g03430); G-H: β-Fructosidase -invertase (VIT_00s2527g00010); I-J: Flavonoid 3’5’-hydroxylase (VIT_06s0009g02910); K-L: UDP-glucose:flavonoid glucosyltransferase (VIT_04s0023g01290). [file 1471-2164-12-631-S3.PDF]
